# Small near-infrared photochromic protein for photoacoustic multi-contrast imaging and detection of protein interactions in vivo

**DOI:** 10.1038/s41467-018-05231-3

**Published:** 2018-07-16

**Authors:** Lei Li, Anton A. Shemetov, Mikhail Baloban, Peng Hu, Liren Zhu, Daria M. Shcherbakova, Ruiying Zhang, Junhui Shi, Junjie Yao, Lihong V. Wang, Vladislav V. Verkhusha

**Affiliations:** 1 Caltech Optical Imaging Laboratory, Department of Electrical Engineering, California Institute of Technology, Pasadena, CA 91125 USA; 2 Department of Anatomy and Structural Biology, and Gruss-Lipper Biophotonics Center, Albert Einstein College of Medicine, Bronx, NY 10461 USA; 3 Department of Biomedical Engineering, Washington University in St. Louis, St. Louis, MO 63130 USA; 4 Caltech Optical Imaging Laboratory, Andrew and Peggy Cherng Department of Medical Engineering, California Institute of Technology, Pasadena, CA 91125 USA; 5 Department of Biomedical Engineering, Duke University, Durham, NC 27708 USA; 6 Medicum, Faculty of Medicine, University of Helsinki, 00290 Helsinki, Finland

## Abstract

Photoacoustic (PA) computed tomography (PACT) benefits from genetically encoded probes with photochromic behavior, which dramatically increase detection sensitivity and specificity through photoswitching and differential imaging. Starting with a DrBphP bacterial phytochrome, we have engineered a near-infrared photochromic probe, DrBphP-PCM, which is superior to the full-length RpBphP1 phytochrome previously used in differential PACT. DrBphP-PCM has a smaller size, better folding, and higher photoswitching contrast. We have imaged both DrBphP-PCM and RpBphP1 simultaneously on the basis of their unique signal decay characteristics, using a reversibly switchable single-impulse panoramic PACT (RS-SIP-PACT) with a single wavelength excitation. The simple structural organization of DrBphP-PCM allows engineering a bimolecular PA complementation reporter, a split version of DrBphP-PCM, termed DrSplit. DrSplit enables PA detection of protein–protein interactions in deep-seated mouse tumors and livers, achieving 125-µm spatial resolution and 530-cell sensitivity in vivo. The combination of RS-SIP-PACT with DrBphP-PCM and DrSplit holds great potential for noninvasive multi-contrast deep-tissue functional imaging.

## Abstract (web summary)

Bacterial phytochrome-based probes improved sensitivity in photoacoustic computed tomography. Here the authors engineer a small near-infrared switchable photochromic probe that allows multi-contrast imaging at depths and can be adapted to study protein–protein interactions in deep-seated tumors.

## Introduction

To better understand the molecular mechanisms and dynamics involved in physiology and disease in a whole organism, biomedical studies increasingly employ noninvasive whole-body imaging with high-resolution in vivo^1–3^. Optical imaging offers valuable information and has been widely used in such studies^4,5^. However, photons are strongly scattered in biological tissue, limiting high-resolution pure optical imaging to a penetration depth within the optical diffusion limit (~1 mm)^6^. Photoacoustic (PA) computed tomography (PACT), by acoustically detecting photons absorbed by tissue, breaks the resolution and depth limitations of pure optical imaging and provides high-resolution imaging with optical contrast at depths up to centimeters^7^. PACT, highly sensitive to optical absorption by molecules, is inherently suited for molecular imaging using optically absorbing probes^8–12^.

Genetically encoded probes are advantageous due to their harmless non-invasiveness, precisely controllable targeting, and tissue-specific promoters. The combination of PACT and a reversibly photoswitchable near-infrared (NIR) absorbing full-length bacterial phytochrome (BphP) from *Rhodopseudomonas palustris*, RpBphP1, has resulted in an advanced differential imaging technique, termed reversibly switchable PACT (RS-PACT). RS-PACT provided substantially enhanced detection sensitivity in deep tissues^13^ in comparison with conventional PACT. PACT is now widely used with various proteins exhibiting reversible photochromic behavior^14^. Temporal unmixing has been applied to separate signals from two photoswitchable fluorescent proteins (FPs) in tissue phantoms^15,16^, however, their short absorption wavelengths make them less suited to deep-tissue PA imaging. Dual wavelength excitation has also been proposed to improve imaging sensitivity of BphPs, but is still limited to detecting only one phytochrome in vivo^17,18^.

**Fig. 1:**
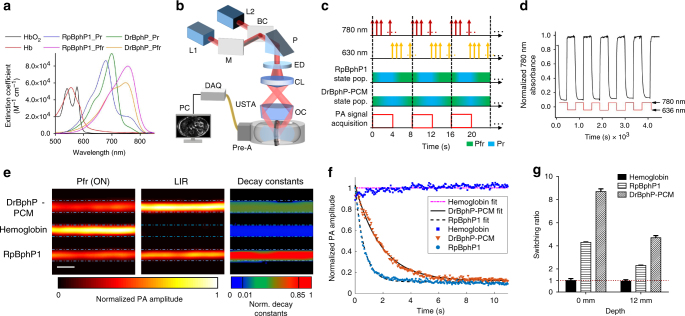
Spectral and photoacoustic characterization of the DrBphP-PCM. **a** Molar extinction spectra of oxy-hemoglobin (HbO_2_), deoxy-hemoglobin (Hb), Pfr (ON), and Pr (OFF) state of DrBphP-PCM and RpBphP1. **b** Schematic of the whole-body photoacoustic computed tomography (PACT) system with a ring-shaped illumination pattern. BC beam combiner, CL conical lens, DAQ data acquisition unit, ED engineering diffuser, M mirror, OC optical condenser, P prism, PC personal computer, pre-A pre-amplifier, USTA ultrasonic transducer array. L1, a Ti:Sapphire laser fired at 780 nm is used for PA imaging and switching off BphPs. L2, the optical parametric oscillator (OPO) laser, fired at 630 nm, switches BphPs ON. **c** Time sequence of photoswitching and imaging of BphPs (pop., population). **d** Absorbance of DrBphP-PCM at 780 nm, switched OFF with 780 nm light illumination and then switched ON with 630 nm illumination. The photoswitching period was 180 s for both wavelengths. **e** PA images of transparent silicone tubes filled with proteins in clear media. Left column: ON state PA image of BphPs and hemoglobin, middle column: frequency lock-in reconstructed (LIR) PA image of BphPs and hemoglobin; right column, decay constant encoded image showing a reliable separation of DrBphP-PCM, RpBphP1, and non-switchable hemoglobin. Scale bar, 500 µm. **f** PA signal changes upon 780 nm light illumination and their fits, where PA signals from either DrBphP-PCM or RpBphP1 exponentially decrease during photoswitching, but with different decay constants. However, the blood signal remains at the original level. Thus, the difference in decay constants enables a good separation of hemoglobin, DrBphP-PCM, and RpBphP1. **g** The switching ratio of BphPs and hemoglobin, defined as the ratio between the ON and OFF states of the PA amplitude, in both clear medium (0 mm in depth) and scattering medium (12 mm in depth); error bars are s.e.m. (*n* = 40), calculated based on the pixel values from regions of interest

Structurally, BphP proteins consist of a photosensory core module (PCM) and various so-called effector domains^19–21^ (Supplementary Fig. 1). The PCM is formed by the PAS (Per-ARNT-Sim), GAF (cGMP phosphodiesterase/adenylate cyclase/FhlA), and PHY (phytochrome-specific) protein domains connected with α-helix linkers, and typically has a molecular weight of 55–58 kDa. The spectral properties of BphPs are determined by a covalently attached chromophore, biliverdin IXα (BV). BV is an enzymatic product of heme and is widely present in mammalian cells and tissues. Incorporation of BV by an apoform of the BphP protein occurs in two steps. First, BV is secured to a chromophore-binding pocket in the GAF domain, and second, a covalent thioether bond is formed between the pyrrole ring A (C3^2^ atom) and the cysteine residues in the PAS domain^22,23^ (Supplementary Fig. 1). Canonical natural BphPs have two absorbing states, one of which absorbs at 670–700 nm (the Pr state) and the other at 740–780 nm (the Pfr state) (Fig. 1a). All BphPs exhibit natural photochromic behavior: they undergo reversible Pfr → Pr photoswitching upon 730–790 nm light irradiation and Pr → Pfr photoswitching upon 630–690 nm light irradiation. Here, we term the Pfr state of the BphP-based probes the ON state and the Pr state the OFF state.

The RpBphP1, used in RS-PACT, consists of the PCM and two additional effector domains, named the PAS/PAC and HOS domains, and forms a dimer^24^. Moreover, the HOS domain of one monomer interacts with the PCM of another monomer in the dimer. Because of its high molecular weight of ~82 kDa, RpBphP1 exhibits a limited folding efficiency and low expression level in mammalian cells. Attempts to delete the HOS domain resulted in the loss of photochromic behavior, suggesting that HOS binding to PCM is required for photoswitching. This finding was surprising, because in some BphPs, deletion of the effector domains does not affect reversible photoswitching, and only further truncation of the PHY domain starts to impair it^25,26^. A PCM part of the DrBphP phytochrome from *Deinococcus radiodurans* (termed DrBphP-PCM below) does not interact with effector domains, preserves photochromism without effector domains, and is 1.5 times smaller than RpBphP1^27^ (Supplementary Fig. 1). These features make it an attractive template for engineering advanced PA probes.

Currently, because of the absence of PA probes with NIR absorbance, whole-body molecular imaging of protein–protein interactions (PPIs) employs bioluminescent luciferases and FPs. PPI studies utilize Förster resonance energy transfer (FRET), bioluminescence energy transfer (BRET), and bimolecular fluorescence complementation (BiFC) approaches. However, relatively small changes in the FRET and BRET signals make these techniques suboptimal for use in whole mammals. BiFC is based on the tagging of two proteins of interest, each with half of an FP. Upon interaction of the proteins, the two halves of the split FP associate with each other to form a fluorescent complex with the complemented FP, thus reporting the PPIs. Recently, we engineered several BiFC reporters from NIR FPs and demonstrated their ability to detect PPIs in mice^28,29^. However, NIR BiFC did not provide high spatial resolution and sensitivity in imaging PPIs in deep tumors. PPIs were also imaged in vivo using split luciferase^30–33^ and thymidine kinase^34^, resulting in bioluminescence and positron emission signals, respectively. However, these reporters require injection of substrates. Moreover, the emission of the most red-shifted split luciferase is limited to 615 nm^33^, and thymidine kinase’s signal provides low contrast and a non-specific background in vivo.

Here, we report a PACT technique which combines three approaches, namely single-impulse panoramic PACT (SIP-PACT)^2^, RS-PACT^13^, and real-time detection of the photoswitching rates of genetically encoded photochromic probes. We term this combined technique RS-SIP-PACT. We also characterize DrBphP-PCM both in vitro and in vivo as an advanced NIR photochromic probe for PACT techniques and demonstrate that it outperforms RpBphP1. We introduce both BphPs into the same mammalian cells, resulting in a distinctive decay characteristic in comparison with the cells expressing DrBphP-PCM only. By discriminating the different decay characteristics, we successfully separate both cell types in deep tissue. Using a single illumination wavelength, we perform multi-contrast temporal frequency lock-in PA reconstruction (LIR) of two different tumors expressing the BphPs at depths in vivo. We next engineer a split version of DrBphP-PCM, resulting in the first bimolecular photoacoustic complementation (BiPC) reporter, termed DrSplit, and apply it to study intracellular PPIs in deep-seated mouse tumors and livers in vivo.

## Results

### Design and characterization of RS-SIP-PACT system

To characterize DrBphP-PCM as a PA probe and compare it with RpBphP1, we upgraded SIP-PACT for real-time reversible photoswitching, detection of photoswitching rates, and imaging, creating RS-SIP-PACT, which provides 125 µm in-plane resolution and ~1 mm elevational resolution (Supplementary Fig. 2). In order to image RpBphP1, DrBphP-PCM proteins, and the DrSplit reporter, we combined a Ti:Sapphire laser and an optical parametric oscillator (OPO) for illumination. These two lasers were synchronized and triggered by an FPGA-based controller (Methods). While the previous RS-PACT took 1.6 s to form a cross-sectional image with eight times multiplexing^13^, RS-SIP-PACT requires only 50 µs to acquire data for one frame with a single laser pulse. Moreover, although its frame rate is currently limited by the imaging laser’s repetition rate (20 Hz), RS-SIP-PACT has achieved a 32-times greater frame rate than the previous RS-PACT^13^. Due to the high-imaging speed of RS-SIP-PACT, we are able to capture the entire photoswitching process of the BphPs in real time, which enables temporal frequency analysis on each pixel. The result is a better contrast-to-noise ratio (CNR) in the images of BphPs, and a reduction in the impacts of motion (e.g., from respiration and heart beating) during in vivo imaging. In addition, the real-time detection of the photoswitching rates of BphPs allows a good separation of RpBphP1 and DrBphP-PCM, which have different photoswitching rates, using a single wavelength excitation.

### Comparison of DrBphP-PCM and RpBphP1 as PA probes

We first measured the molar extinction coefficients for the ON states and the OFF states of DrBphP-PCM and RpBphP1. The ratios between the extinction coefficients of the ON state (Pfr form) at 780 nm and the OFF state (Pr form) at 630 nm of DrBphP-PCM and RpBphP1 were 9.9 and 4.1, respectively (Fig. 1a and Supplementary Fig. 3). We employed 780 nm light for PA imaging and photoswitching the BphPs to the OFF state, and used 630 nm light to switch the BphPs back to the ON state (Fig. 1b, c). The laser fluence on the sample surface at both wavelengths did not exceed 12 mJ cm^−2^, which is below the American National Standards Institute safety limit^35^. The imaging and photoswitching time sequences are shown in Fig. 1c. The change in RpBphP1 absorbance at 780 nm between the ON and OFF states was about four times, similar to earlier observations^13^. The changes in DrBphP-PCM absorbance at 780 nm between the ON and OFF states were two times larger than that of RpBphP1 (Fig. 1d), which resulted in higher PA imaging contrast (Supplementary Table 1).

Tubes filled with DrBphP-PCM (~30 µM), hemoglobin (bovine blood with 90% oxygen saturation, sO_2_), and RpBphP1 (~30 µM), respectively, were first embedded in clear gelatin (Fig. 1e). Although hemoglobin has the highest contrast in the ON state images (Fig. 1e, left column), in LIR, where a pixel-wise extraction of amplitudes of the harmonics of the illumination modulation frequency, both DrBphP-PCM and RpBphP1 signals stand out (Fig. 1e, middle column). The LIR method successfully separated the PA signals from two BphPs from the non-photoswitchable blood signals, even with 2.5 times higher CNR than previous differential method^13^ (Methods and Supplementary Fig. 4). Typically, a threshold level of four times the noise level, estimated as the standard deviation of the background signal outside the imaged region, was globally applied to the PA LIR images.

Compared to RpBphP1, DrBphP-PCM took about three times longer time to photoswitch from the ON state to the OFF state (Fig. 1f, Supplementary Fig. 3c–f, and Supplementary Table 1). This photochemical feature enabled separating the PA signals of DrBphP-PCM and RpBphP1 by measuring the signal decay constants during imaging. Moreover, since hemoglobin is non-photoswitchable, its decay constant was close to zero, making it even more distinguishable from the BphP-based probes in RS-SIP-PACT (Fig. 1e, right column). The ON-to-OFF photoswitching rate (decay constant) here is defined as the reciprocal of the time it takes for the PA signal from the protein to drop to 1/*e* of its maximum. The ON-to-OFF photoswitching rates of DrBphP-PCM and RpBphP1 were 0.54 s^–1^ and 1.56 s^–1^, respectively, as measured at a laser fluence of 4 mJ cm^–2^ at 780 nm (Supplementary Table 1).

We further compared the reversible photoswitching of both BphPs in scattering media at depths using 780 nm illumination. Tubes filled as before were embedded at a depth of 12 mm inside a scattering medium (10% gelatin and 1% intralipid in distilled water; reduced scattering coefficient of ~10 cm^−1^)^36^. We defined the photoswitching ratio as the ratio of the measured PA signal amplitude of BphPs in the ON state to that in the OFF state. In both the clear medium (0 mm in depth) and scattering medium (12 mm in depth), DrBphP-PCM exhibited two times better photoswitching ratio than RpBphP1 (Fig. 1g).

### Multi-contrast RS-SIP-PACT imaging in cells and in vivo

To compare expression levels of DrBphP-PCM and RpBphP1 in mammalian cells, we designed two similar plasmids where EGFP was co-expressed through a self-cleavable T2A peptide after the BphPs. The expression from these plasmids resulted in equimolar levels of a BphP and an EGFP control. Flow cytometry of HeLa cells transfected with these plasmids showed that the DrBphP-PCM expression level was 2.3 times higher than that of RpBphP1 (Supplementary Fig. 5a, b), likely because of the 1.5 times smaller size and simpler structural organization of the DrBphP-PCM, which enabled faster protein folding at 37 °C in mammalian cells.

**Fig. 2:**
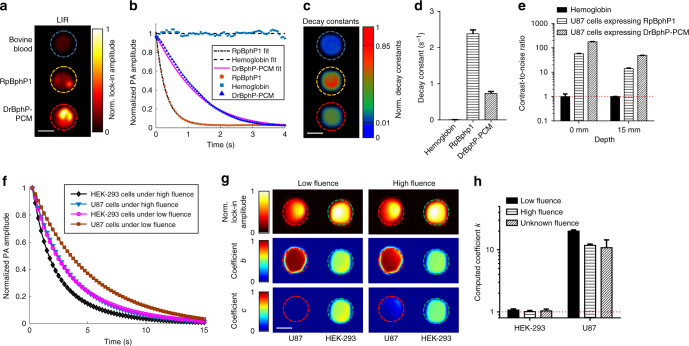
Photoacoustic characterization of the BphPs in cultured cells. **a** LIR image of bovine blood, U87 cells expressing either RpBphP1 or DrBphP-PCM. Scale bar, 2 mm. **b** PA signal decays and their fits from bovine blood, U87 cells expressing either RpBphP1 or DrBphP-PCM during 780 nm light illumination. **c** Decay constant encoded image showing different photoswitching rates of U87 cells expressing either RpBphP1 or DrBphP-PCM and the non-switchable bovine blood. Scale bar, 2 mm. **d** The computed decay constants of the three types of cells; error bars are s.e.m. (*n* = 40), calculated based on the pixel values from regions of interest. **e** The contrast-to-noise ratio (CNR) of LIR signals from bovine blood and from U87 cells expressing either RpBphP1 or DrBphP-PCM in a clear medium (0 mm in depth) and a scattering medium (15 mm in depth). **f** PA signal decays and their fits for the two types of cells—HEK-293 cells expressing both DrBphP-PCM and RpBphP1, and U87 cells expressing only DrBphP-PCM—under different illumination fluences. **g** LIR images (top row) of U87 cells (left) and HEK-293 cells (right) and the images of computed coefficients of *b* (middle row) and *c* (bottom row) under different illumination fluences. The signal decays can be modeled in the form of $g(t) = a + b \cdot e^{( {\frac{{ - t}}{{T_1}}})} + c \cdot e^{( {\frac{{ - t}}{{T_2}}})}$, where *T*_1_ > *T*_2_. The signals from HEK-293 cells were fitted with two similar coefficients *b* ≈ *c* ≈ 0.5, while the signals from U87 cells were fitted with very different coefficients *b* ≈ 1, *c* ≈ 0. Scale bar, 2 mm. **h** The computed coefficients *k*, defined as $k = \frac{{{\rm{max}}\{ b,c\} }}{{{\rm{min}}\{ b,c\} }}$, under different light fluences, showing a reliable separation of the two types of cells in **f**, **g**. Independent of the light fluence, the coefficient *k* for HEK-293 cells is ~1, and the coefficient *k* for U87 cells is much larger (>10); error bars are s.e.m. (*n* = 120), calculated based on the pixel values from regions of interest

We next used RS-SIP-PACT to image U87 glioblastoma cells stably expressing either RpBphP1 or DrBphP-PCM. With the RS-SIP-PACT system, we imaged pure bovine blood and either RpBphP1 or DrBphP-PCM expressing U87 cells embedded in gelatin (Fig. 2a, Supplementary Fig. 5c, and Supplementary Movie 1). The decay constants from the ON to the OFF state enabled good separation of the blood and both types of U87 cells (Fig. 2b–d and Supplementary Fig. 5d).

Blood and U87 mammalian cells were then embedded 15 mm deep in a scattering medium with a reduced scattering coefficient of ~10 cm^−1^. We imaged the cells for ten photoswitching cycles, each cycle containing 80 frames, and selectively distinguished the PA signals from BphPs by using LIR (Methods). U87 cells expressing DrBphP-PCM had CNRs of 176.7 ± 6.8 in the clear medium and 48.4 ± 3.6 in the scattering medium. However, U87 cells expressing RpBphP1 had lower CNRs of 56.9 ± 5.8 and 14.0 ± 1.5, respectively (Fig. 2e). The higher DrBphP-PCM expression level, together with the higher absorbance ratio between the photoswitching states, resulted in a three–four times enhancement of CNR for DrBphP-PCM over that of RpBphP1 in the cultured mammalian cells. Moreover, in comparison to previous differential imaging technique, LIR provided an approximately two–three times improvement in CNR (Supplementary Fig. 6). Thus, the combination of the DrBphP-PCM and LIR algorithm enabled a ~10 times (=3–4 × 2–3) CNR enhancement over the combination of RpBphP1 and differential imaging, in total (Supplementary Fig. 6).

The decay constants of the photoswitching processes depend on the local fluence. Under similar light fluence, DrBphP-PCM has a longer decay time than RpBphP1. However, due to optical absorption and scattering, the local optical energy delivery per unit area is unknown inside deep tissue, which poses a substantial challenge to unmixing multiple contrasts at depths using their decay constants. To address the unknown local optical fluence, we proposed a labeling strategy where we introduced both DrBphP-PCM and RpBphP1 into the same HEK-293 mammalian cells and introduced DrBphP-PCM only into U87 cells. Thus, the HEK-293 cells exhibited a distinctive decay characteristic in comparison with the U87 cells.

We used RS-SIP-PACT to image the HEK-293 cells expressing both BphPs in equimolar quantities from a single plasmid (Methods) and the U87 cells expressing only DrBphP-PCM. For each measurement voxel, we reasonably assumed that the local fluence was uniform within that voxel, because the 1/*e* optical penetration depth for NIR light is far greater than the voxel length. Experimental results showed that the photoswitching signals from HEK-293 cells expressing both BphPs contained two decay components, while the signals from U87 cells expressing DrBphP-PCM exhibited only one decay component, regardless of local fluence (Fig. 2f, Supplementary Fig. 7a–d, and Supplementary Table 2). With this labeling strategy, we took advantage of the number of decay components involved in the decay process to reliably separate two types of cells, instead of relying on the decay rates.

We modeled the decay process as a linear combination of two single exponential decay functions with different decay constants (Methods). By comparing the contributions of both decay functions to the overall decay, we established a criterion: If the contribution from the slower decay process is significantly larger (10×) than that from the faster decay process, the decay process is dominated by one component and the signals are from U87 cells; if the two contributions are similar (a ratio of ~1), the number of decay components is two and the signals are from HEK-293 cells. Therefore, by computing the number of decay components involved in the decay process, we can reliably separate the two types of cells in deep tissue, where knowledge of local fluence is limited (Fig. 2g, h, Supplementary Fig. 7e, and Supplementary Table 2). If the HEK-293 and U87 cells are mixed together and cannot be spatially resolved, we can quantify the concentration of each cell type by comparing the contributions from the two decay functions (Methods, Supplementary Fig. 7f, and Supplementary Table 2).

**Fig. 3:**
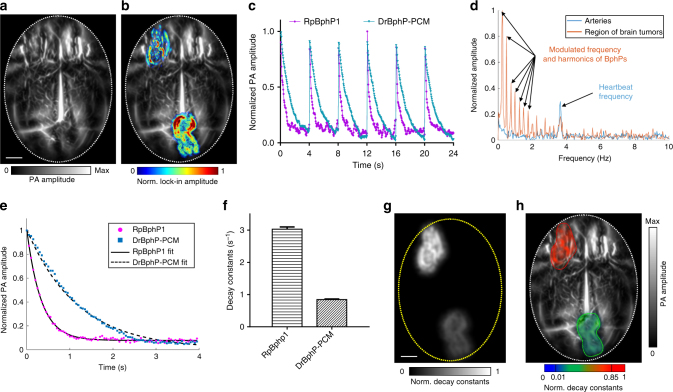
Multi-contrast PA imaging of BphPs in the mouse brain in vivo. **a** Conventional PA image of the tumor-bearing mouse brain cortex vasculature (ON state). Approximately 1 × 10^6^ U87 cells expressing DrBphP-PCM were injected into the left front of the brain, ~1 × 10^6^ U87 cells expressing RpBphP1 were injected into the right rear of the brain. The tumors are invisible in the ON state images due to the overwhelming background signals from blood. Scale bar, 2 mm. **b** LIR image overlaid on the mouse brain cortex vasculature, highlighting the two tumors of U87 cells expressing either RpBphP1 or DrBphP-PCM. The overlay image shows the BphP signals in color and the background blood signals in gray. **c** PA signals from two tumors were modulated at the same frequency by the illumination but with different signal decay constants. **d** Temporal frequency spectra of the PA signals from brain tumors and the cortical arteries, showing both the harmonics of the illumination modulation frequency and the heartbeat frequency from the arteries. **e** PA signal decays and their fits for the two tumors expressing either DrBphP-PCM or RpBphP1. **f** The computed decay constants of the two tumors; error bars are s.e.m. (*n* = 160), calculated based on the pixel values from regions of interest. **g** Decay constant encoded image illustrating good separation of the two tumors. Scale bar, 2 mm. **h** Decay constant encoded image overlaid on the mouse brain cortical vasculature, showing reliable separation of two tumors

**Fig. 4:**
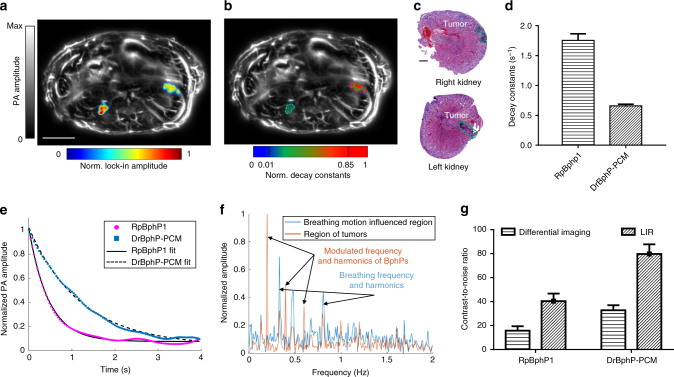
In vivo lock-in reconstruction of BphPs in kidneys and their decay analysis. **a** LIR image overlaid on a conventional PACT cross-sectional image by 780 nm illumination, highlighting the two tumors of U87 cells expressing either RpBphP1 (right side) or DrBphP-PCM (left side) on the two kidneys. The overlay image shows the BphPs signal in color and the background blood signal in gray. **b** Decay constant encoded image overlaid on a conventional PACT cross-sectional image made by 780 nm illumination. The LIR image was used to form a binary mask, and the decay constant computation was implemented in the masked regions. **c** Representative H&E histological images of the two isolated kidneys, showing the tumors (bordered by green lines) corresponding to **a** and **b**. Scale bar, 1 mm. **d** The computed decay constants of the two tumors. **e** PA signal decays and their fits in the tumor regions. **f** Temporal frequency spectra of the PA signals in the kidney tumors and the internal organs, showing both the harmonics of the illumination modulation frequency and the harmonics of the respiratory frequency. **g** The LIR method provides approximately two–threefold better CNR of tumor cells expressing either DrBphP-PCM or RpBphP1 than the differential imaging method; error bars are s.e.m. (*n* = 80), calculated based on the pixel values from regions of interest. Scale bar, 5 mm

To study the performance of DrBphP-PCM in vivo, we first injected 1 × 10^6^ U87 cells expressing DrBphP-PCM into a mouse brain and successfully detected the tumors using RS-SIP-PACT (Supplementary Fig. 8). To demonstrate the advantages of DrBphP-PCM in vivo, we imaged a mouse 2 weeks after injection of 1 × 10^6^ U87 cells expressing DrBphP-PCM into the left front of the brain and 1 × 10^6^ U87 cells expressing RpBphP1 into the right rear of the brain. A conventional PACT image reveals the brain’s cortical vasculature (Fig. 3a). In addition, LIR of ten photoswitching cycles of conventional PACT images (each cycle containing 80 frames) highlights the two U87 tumors (overlaid on the cortex vasculature, Fig. 3b). We cannot directly separate the DrBphP-PCM tumor from the RpBphP1 tumor, because the two tumors have the same modulation frequency (Fig. 3c, d). Because the tumors were seated beneath the scalp tissue, the local fluence values at the two tumors were comparable. We thus could separate them by directly comparing their decay constants (Fig. 3e–h and Supplementary Movie 2). We also demonstrated this separation in peripheral regions of mouse kidneys (Fig. 4 and Supplementary Movie 3). After PA imaging, we confirmed the tumors histologically (Fig. 4c)

The previous RS-PACT system required 1.6 s to form a cross-sectional image, which blurred whole-body images due to respiratory motion during data acquisition and thus reduced the detection sensitivity. In comparison, RS-SIP-PACT takes just 50 µs to form a cross-sectional image, with completely negligible motion artifacts. By taking advantage of this real-time imaging capability, RS-SIP-PACT can image the decays of both BphPs while monitoring the respiratory motion. Thus, LIR can highlight the tumors, with minimized motion artifacts and high contrast (Fig. 4a, f, g).

**Fig. 5:**
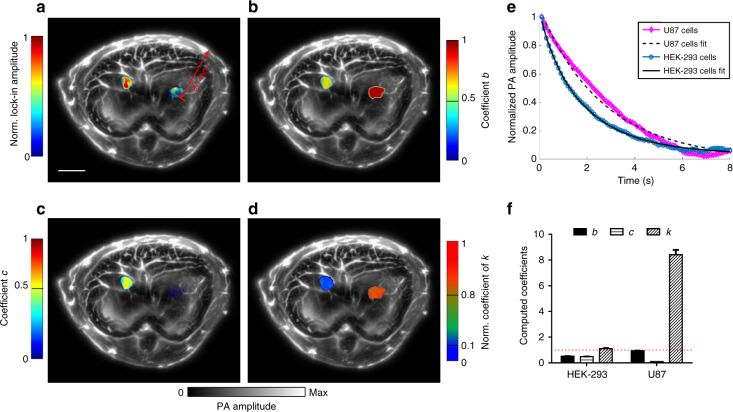
In vivo separation of two types of cells at depths. The PA excitation wavelength was 780 nm. **a** LIR image overlaid on a conventional PACT cross-sectional image by 780 nm illumination, highlighting the two tumors of HEK-293 cells expressing both DrBphP-PCM and RpBphP1 (left lobe) or U87 cells expressing DrBphP-PCM (right lobe) inside the liver (*n* = 3). The overlay image shows the BphP signals in color and the background blood signals in gray. **b** Coefficient *b* encoded image overlaid on a conventional PACT cross-sectional image. The computed coefficient, *b*, is shown in color, and the background anatomy is shown in gray. The LIR image in **a** was used to form a binary mask, and the decay analysis was implemented in the masked regions. **c** Coefficient *c* encoded image overlaid on a conventional PACT cross-sectional image. The computed coefficient, *c*, is shown in color, and the background anatomy is shown in gray. **d** Normalized coefficient *k* encoded image overlaid on a conventional PACT cross-sectional image. Because the HEK-293 tumors contain two different photochromic proteins and U87 tumors contain only one photochromic protein, the normalized coefficient *k* of HEK-293 tumors is much smaller than that of U87 tumor, showing a reliable separation of the two tumors. The LIR image was used to form a binary mask, and the decay constant computation was implemented in the masked regions. **e** PA signal decays and their fits in the tumor regions. **f** The computed coefficients of *b*, *c*, and *k* from the tumor regions, where *k*, showing the largest difference, can be used to separate the two types of tumors. Independent of the light fluence, the coefficient *k* for HEK-293 tumors is ~1, and the coefficient *k* for U87 tumors is much larger (>8); error bars are s.e.m. (*n* = 140), calculated based on the pixel values from regions of interest. Scale bar, 5 mm

To reliably separate the two types of tumors inside deep tissue in vivo, we applied the same labeling strategy to mouse liver tumors. We first injected U87 cells expressing DrBphP-PCM (0.5 × 10^6^) into the right lobe of the mouse liver and waited 5 days to allow the injected U87 cells to grow. After the waiting period, we injected HEK-293 cells expressing both BphPs (8 × 10^6^) into the left lobe of the liver. At 2 h post injection, we then imaged the tumor-bearing mouse (*n* = 3) for 20 photoswitching cycles, each of which contained 160 frames. The LIR image clearly resolved the two tumors, with minimized motion artifacts (Fig. 5a). The HEK-293 tumors contain two different photochromic proteins, exhibiting two different decay constants in the decay process (Fig. 5b, c); while the U87 tumors contain only one photochromic protein, exhibiting only one decay constant in the decay process (Fig. 5b, c). Moreover, by analyzing the number of decay constants involved, we achieved reliable differentiation between the two tumors in deep tissue (~9.1 mm beneath skin, Fig. 5d–f).

### Characterization of DrSplit for protein–protein interaction

**Fig. 6:**
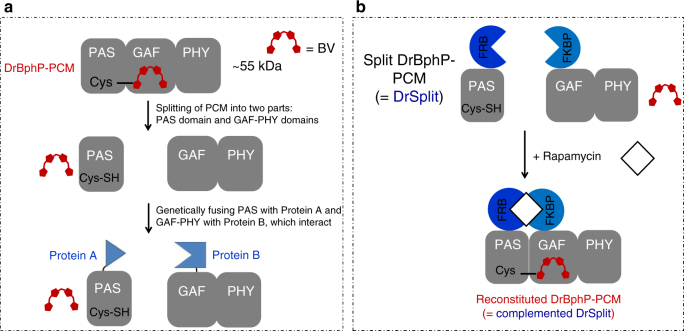
Development of the bimolecular photoacoustic complementation (BiPC) reporter DrSplit. **a** DrBphP-PCM consists of three domains, PAS, GAF, and PHY. The biliverdin (BV) chromophore is covalently bound with conservative cysteine from the PAS domain and secured to a chromophore-binding pocket in the GAF domain. DrBphP-PCM was genetically split into two parts, the PAS domain and GAF-PHY domain, together named DrSplit. In this case, BV does not bind with any part of DrSplit. Genetically fusing one protein of interest (protein A) to one part of DrSplit and another protein of interest (protein B) to another part of DrSplit makes possible the monitoring of protein–protein interactions (PPIs) between protein A and protein B. **b** We used a model rapamycin-induced PPI between the FRB and FKBP proteins for evaluation of DrSplit. FRB was fused to the PAS domain and FKBP was fused to the GAF-PHY domains. Upon addition of rapamycin to the DrSplit, DrBphP-PCM was re-functionalized

We next designed a BiPC reporter from DrBphP-PCM. For this, we genetically separated (split) DrBphP-PCM between the DrPAS domain and the DrGAF-PHY domains, and termed the set of these two constructs DrSplit (Fig. 6a). Notably, the PAS-GAF domains alone do not exhibit reversible photoswitching^37^. Complementation of the PAS domain with the GAF-PHY domain reconstitutes the complete PCM (i.e., PAS-GAF-PHY domains), thus recovering its photoswitching property. To test DrSplit complementation, we used a rapamycin-induced PPIs between the FRB and FKBP proteins^28,29^. We genetically fused the FRB protein to the DrPAS domain, and the FKBP protein to the DrGAF-PHY domains (Fig. 6b).

**Fig. 7:**
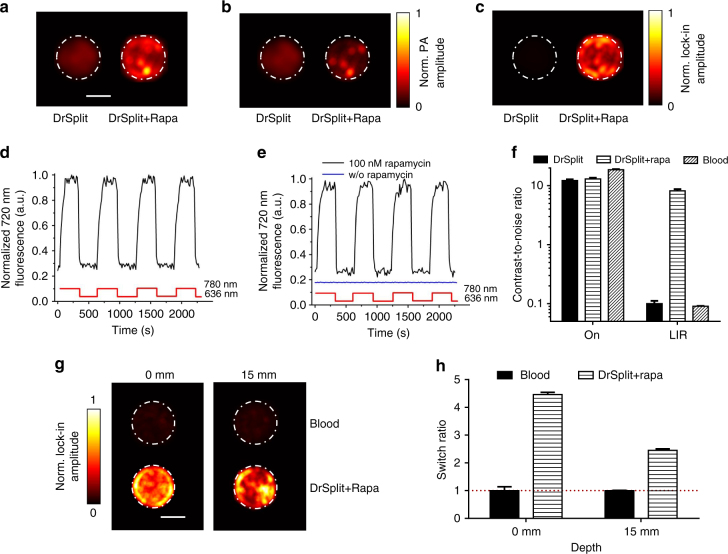
PA characterization of DrSplit in mammalian cells. **a** ON state PA image of MTLn3 cells expressing DrSplit (left) and MTLn3 cells expressing DrSplit in the presence of rapamycin (right). Scale bar, 2 mm. **b** OFF state PA image of MTLn3 cells expressing DrSplit (left) and MTLn3 cells expressing DrSplit in the presence of rapamycin (right). **c** LIR PA image of MTLn3 cells expressing DrSplit (left) and MTLn3 cells expressing DrSplit in the presence of rapamycin (right). The induction with rapamycin reconstitutes the functional DrBphP-PCM, which responds to the periodical light modulation. **d** Repeated fluorescence changes of the lysate of HeLa cells expressing DrBphP-PCM detected at 720 nm during recurrent illumination cycles with 780/20 nm and 636/20 nm. **e** Repeated fluorescence changes of the lysate of HeLa cells expressing DrSplit in the presence (black line) or absence (blue line) of rapamycin, detected at 720 nm during recurrent illumination cycles with 780/20 nm and 636/20 nm. **f** MTLn3 cells expressing DrSplit and MTLn3 cells expressing DrSplit in the presence of rapamycin and blood (dilute 10×) show similar CNRs in the ON state PA image; while the LIR image shows an outstanding CNR for MTLn3 cells expressing DrSplit in the presence of rapamycin. **g** LIR image of blood and MTLn3 cells expressing DrSplit in the presence of rapamycin in a clear medium (0 mm in depth) and a scattering medium (15 mm in depth). Scale bar, 2 mm. **h** The switching ratio of blood and MTLn3 cells expressing DrSplit in the presence of rapamycin in both a clear medium (0 mm in depth) and a scattering medium (15 mm in depth); error bars are s.e.m. (*n* = 40), calculated based on the pixel values from regions of interest. Rapa is short for rapamycin

Because the main application of DrSplit was foreseen as in vivo imaging (see below), we made MTLn3 breast adenocarcinoma cells stably co-expressing DrPAS-FRB and FKBP-DrGAF-PHY fusions and studied their complementation using RS-SIP-PACT. Upon addition of rapamycin to the cells, a BiPC occurred, reconstituting the functional DrBphP-PCM (Fig. 7a–c). Complemented DrSplit retained 36% of the Pr state fluorescence of the non-split DrBphP-PCM (Supplementary Fig. 9a) and almost 100% of Pr ↔ Pfr photoswitching contrast measured by changes of intrinsic fluorescence (Fig. 7d, e). And the Pr ↔ Pfr photoswitching contrast measured by PA is retained ~50% (Supplementary Fig. 9b–d). DrSplit photoswitching at 780 nm was practically non-detectable without rapamycin, but was restored after FRB-FKBP binding induced by rapamycin, thus providing four times contrast (Supplementary Fig. 9d). The CNRs of the LIR images in the absence or presence of rapamycin were ~0.1 and 8.2, representing an ~82 times change in the CNRs of LIR images upon PPI induction (Fig. 7f). Complemented DrSplit in MTLn3 cells exhibited photoswitching ratios of 4.46 ± 0.49 in the clear medium and 2.60 ± 0.34 in the scattering medium (Fig. 7g, h). We then analyzed the expression of DrSplit in HeLa and U87 cells, and found that we could detect the fluorescence of EGFP co-expressed with DrPAS-FRB and mCherry co-expressed with FKRB-DrGAF-PHY, even 72 h after the transfection, indicating a low cytotoxity of the DrSplit (Supplementary Fig. 10).

We have also demonstrated DrSplit’s application for microscopic imaging (Supplementary Fig. 11). MTLn3 cells, stably expressing DrPAS-FRB with EGFP and FKRB-DrGAF-PHY with mCherry, exhibited fluorescence of co-expressed fluorescent proteins in the absence of rapamycin. Upon induction of PPIs with rapamycin, we detected weak intrinsic NIR fluorescence of the DrBphP-PCM reconstituted from DrSplit (Supplementary Fig. 11a, b). The complementation of the DrSplit reporter provided approximately ten-time stronger fluorescence in the NIR channel (Supplementary Fig. 11c). Thus, DrSplit can be used as a multimodal reporter not only for BiPC, but also for BiFC.

### RS-SIP-PACT imaging of PPIs in vivo with DrSplit

**Fig. 8:**
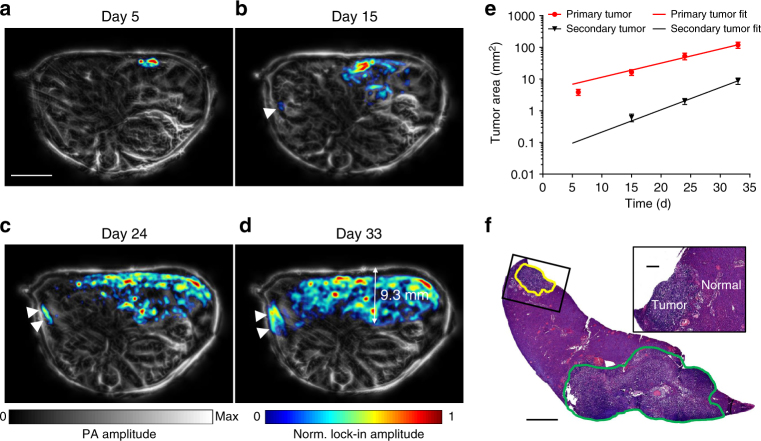
Longitudinal imaging of PPIs in a tumor and monitoring of tumor metastases in a mouse liver. Approximately 1 × 10^6^ MTLn3 cells expressing DrSplit were injected into the mouse liver. The mice (*n* = 4) were imaged at multiple time points after tumor cell injection, and rapamycin was injected via the tail vein ~40–44 h before each PA imaging. **a**–**d** PA images of the mouse on **a** day 5, **b** day 15, **c** day 24, and **d** day 33 after injection of tumor cells, where the white arrows indicate the secondary tumor. LIR images are overlaid on the anatomical images. The overlay image shows the DrSplit signal in color and the background blood signal in gray. Scale bar, 5 mm. **e** Tumor growth curve, in-plane tumor area vs. time (quantified from LIR images). Error bars represent s.e.m. for results from four animals. **f** A representative H&E histological image of a harvested left lobe of a tumorous liver, showing the tumor metastasis, where the primary tumor and secondary tumor are bordered by green and yellow lines, respectively. Scale bar, 1 mm. The close-up H&E image shows the secondary tumor, which can be clearly differentiated from normal tissue. Scale bar, 100 µm

Using DrSplit and RS-SIP-PACT, we next longitudinally imaged PPIs in the tumors and monitored tumor metastases in the liver of mice (*n* = 4) (Fig. 8a–d, Supplementary Fig. 12, and Supplementary Movie 4). DrSplit-expressing MTLn3 cells (1 × 10^6^) were first locally injected in the mouse liver. Then, rapamycin was injected through the tail vein ~40–44 h before the PA imaging. The LIR PA images highlighted the photoswitchable signals from the complemented DrSplit resulting from the PPIs. We detected exponential growth of the primary tumor in the right lobe of the liver over 1 month (Fig. 8a–e). From day 15, we detected a delayed exponential growth of secondary tumors on the left lobe of the liver, resulted from metastasizing MTLn3 cells spreading to the other liver lobe (Fig. 8b–e). The diameter of the secondary tumor on day 15 was ~400 µm (Fig. 8b). The postmortem histology results confirmed the PA-measured relative locations of the tumors (Fig. 8f). The smallest secondary tumor had a diameter of ~400 µm, assuming that the mean volume of MTLn3 cells is ~2000 µm^3^, each resolution voxel of the secondary tumor contained ~3100 MTLn3 cells. The CNR of the secondary tumor was ~9.7 in the LIR image. At a detection confidence level of 90%, corresponding to a CNR threshold of 1.65, we can detect PPIs with as few as ~530 cells at this depth. We have also demonstrated an application of DrSplit as BiFC reporter at depths in vivo (Supplementary Fig. 13). We detected the fluorescence of reconstituted DrSplit after 32 days of tumor growth upon the induction of PPIs with rapamycin.

**Fig. 9:**
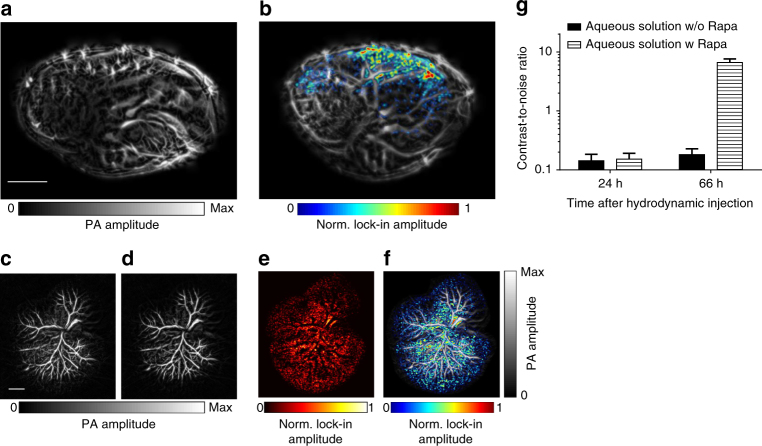
In vivo monitoring of PPIs in a mouse liver after hydrodynamic transfection. **a** PA image of the mouse liver 24 h after hydrodynamic transfection, without induction with rapamycin. The LIR image was overlaid on the anatomical images, but no photoswitching signal was detected. Scale bar, 5 mm. **b** PA image of the mouse liver 42 h after rapamycin injection, with the LIR image overlaid on the anatomical image. **c** Ex vivo PA image (ON state) of the liver excised from the hydrodynamic-transfected mouse. Scale bar, 2 mm. **d** Ex vivo PA image (OFF state) of the excised mouse liver. **e** LIR image of the mouse liver excised from the hydrodynamic-transfected mouse. **f** Overlay of the LIR image and the anatomical image of the excised mouse liver. **g** CNR comparison of the mouse liver region in the LIR image with and without rapamycin injection. Rapamycin was injected through the tail vein 24 h after hydrodynamic liver transfection; Error bars represent s.e.m. for results from four animals. Rapa is short for rapamycin

We next compared non-split DrBphP-PCM and DrSplit in native mouse tissues, without MTLn3 cell transplantation. We first performed hydrodynamic transfection^38^ of the plasmid encoding DrBphP-PCM into the liver of mice (*n* = 4) (Supplementary Figs. 14 and 15). Using RS-SIP-PACT, we found significant changes in the PA signals in the liver-expressing DrBphP-PCM. We then hydrodynamically co-transfected mice with DrPAS-FRB and FKBP-DrGAF-PHY plasmids encoding the DrSplit reporter. As a baseline image, we imaged the mouse liver after 24 h (Fig. 9a). To induce the PPIs resulting in the DrSplit complementation, rapamycin was injected through the tail vein, and the PA signals were detected ~42 h later (~66 h after hydrodynamic injection) (Fig. 9b). Ex vivo PA imaging of the isolated liver from the rapamycin-injected mouse further confirmed the reconstitution of functional DrBphP-PCM from DrSplit (Fig. 9c–f), and the differential fluorescence images also validated the existence of reconstituted DrSplit (Supplementary Fig. 16). The CNRs of the photoswitchable signals in the mouse liver were 0.152 and 6.60 before and after the rapamycin injection, respectively, indicating the ~43 times CNR enhancement resulted from the induced PPIs (Fig. 9g). Upon rapamycin injection, we observed a significant increase of the LIR PA signals, which indicated that PPIs had occurred in the liver tissue, whereas vehicle injection alone did not cause any PA signal changes (Fig. 9g).

## Discussion

PACT is well suited to take maximum advantage of the photochromic behavior of genetically encoded probes. Temporal unmixing for the detection of photoswitchable fluorescent proteins has been demonstrated previously^15,16^. Recently, a BphP from *Agrobacterium tumefacience*, called Agp1^17,18^, was applied for PA imaging in the same manner as RpBphP1 in earlier reported RS-PACT^13^. Here, we combined the advanced RS-SIP-PACT technique with two distinct BphP-based probes, DrBphP-PCM and RpBphP1, which enabled multi-contrast PA in vivo imaging with a single 780 nm excitation. We then designed the first BiPC reporter, DrSplit, and by combining it with RS-SIP-PACT, photoacoustically detected PPIs with high spatial resolution in deep tissues at the whole-body level in mice. These advances resulted from the photochemical and structural features of DrBphP-PCM, which are superior to those of the previously used RpBphP1, as well as from the high-imaging speed and the LIR approach of RS-SIP-PACT, which provided high-sensitivity, high-resolution imaging at depths beyond those achievable by pure optical imaging^39^.

DrBphP-PCM is two times smaller than RpBphP1 and free from interactions between the domains, which facilitates folding and results in its higher expression in mammalian cells. These beneficial differences do not affect cell properties and allow establishing cell lines that stably express DrBphP-PCM. Like RpBphP1, DrBphP-PCM has OFF and ON states in the far-red and NIR regions, where tissues have relatively low-light attenuation, and therefore can maintain its photoswitching efficiency at depths. Further, DrBphP-PCM exhibits a two times greater absorbance photoswitching ratio than RpBphP1^13^. In our experiments, the combination of all these characteristics provided about four times enhancement of the image CNR at depths in vivo. This performance makes DrBphP-PCM the reporter of choice for switching-contrast PACT techniques, such as RS-PACT and the advanced RS-SIP-PACT reported here.

Both DrBphP-PCM and RpBphP1 are photochromic and share similar absorption spectra in the ON and OFF states, so it is not possible to discriminate between these BphPs using standard multi-wavelength unmixing or differential imaging methods. However, DrBphP-PCM has a three-time lower photoswitching rate from the ON to the OFF state than RpBphP1, which requires a longer imaging time to capture the photoswitching process. Considering the unknown fluence, we proposed a labeling strategy where the HEK-293 cells were labeled with both DrBphP-PCM and RpBphP1 in equimolar quantities and U87 cells were labeled with DrBphP-PCM only. By computing the number of decay components involved in the photoswitching process, we successfully separated the two types of cells in deep tissue—a task that, due to unknown local fluence, cannot be reliably done by either the previous RS-PACT^13^ or other temporal unmixing methods^15,16^. Thus, RS-SIP-PACT not only increases the CNR of images by selectively highlighting the photoswitching components using LIR, but also separately detects several spectrally similar photochromic probes based on their unique decay characteristics. Unlike spectral unmixing-based probe separation approaches, probe discrimination based on decay characteristics should allow simultaneous imaging of a substantially larger number of different cells expressing properly designed BphP-derived photochromic probes, using a single wavelength excitation. More importantly, the combination of the decay analysis and the protein development has successfully addressed the impact of unknown local fluence and enabled multi-contrast imaging at depths.

The smaller size and simpler domain interaction in DrBphP-PCM allowed us to design the DrSplit reporter, enabling the BiPC technique to detect PPIs photoacoustically. Compared to the similar, but purely optical, BiFC technique, BiPC provides deeper PPI detection with higher resolution. The BiPC technique introduced here enabled PPI detection in a whole animal with as few as 530 secondary tumor cells. DrSplit, designed in this work, retains most of the advantageous photochromic and PA properties of the parental DrBphP-PCM. Therefore, the BiPC approach should allow simultaneous detection of several PPIs by using multiple BphP-derived split probes that exhibit different ON-to-OFF photoswitching rates. Moreover, unlike BiFC, detection of several PPIs using BiPC can be performed with a single excitation wavelength. Further application of the DrSplit reporter could be simplified by using a self-cleavable peptide, such as T2A, inserted between the DrBphP-PAS and DrBphP-GAF-PHY parts, allowing co-expression of the complete BiPC reporter from a single plasmid.

In turn, the developed RS-SIP-PACT technique, no longer requiring data multiplexing, substantially accelerates the PA imaging process and further improves the detection sensitivity. In addition, the LIR method is simple and reliable; the background signals and respiratory motion influence are removed without loss of spatial resolution and sensitivity.

The NIR photochromic DrBphP-PCM probe and DrSplit PPI reporter engineered here, combined with RS-SIP-PACT, open possibilities in basic biology and biomedical research. Both probes can noninvasively monitor individual pathways in subsets of cells in deep tissue and provide analysis of multiple pathways in a whole organ. DrSplit will allow detection of various biological processes that involve PPIs, such as wound healing, host–pathogen interactions, and organ development, and also serve as a whole-cell sensor for metabolic changes. Although BiPC of split reporters, such as DrSplit, can be irreversible, as with BiFC, it will visualize the accumulation of transient PPIs and low-affinity complexes^40,41^. The higher-detection sensitivity of BiPC can advance the monitoring of activities of drug targets, to identify potential off-target effects by detecting PPIs associated with downstream pathways. Furthermore, it will enable in vivo genome-wide studies of PPIs, which previously were tested with BiFC, outperforming it in depth and spatial resolution^42^.

## Methods

### Photoacoustic tomography

In PAT, as photons propagate in tissue, some are absorbed by biomolecules and their energy is partially or completely converted into heat. The heat-induced pressure propagates in the tissue and is detected outside the tissue by an ultrasonic transducer or transducer array to form an image that maps the original optical energy deposition in the tissue. The scattering of acoustic waves, within the ultrasonic frequency range of interest, is about three orders of magnitude weaker than that of light in soft tissue, on a per unit path length basis, which means that PAT can provide high spatial resolution at depths reachable by diffuse photons. PACT is a major implementation of PAT. A multi-element ultrasonic transducer array, or a mechanical/electronic scanning equivalent, is used to detect photoacoustic waves. Then, an inverse algorithm—essentially a method for accurately locating photoacoustic sources and mapping the absorbed optical energy density from the time-resolved acoustic signals—is employed to reconstruct high-resolution images.

### Plasmid construction

The *DrBphP* gene was kindly provided by J. Ihalainen (University of Jyväskylä, Finland). The *RpBphP1* gene was kindly provided by E. Giraud (Institute for Research and Development, France). For mammalian expression, the *RpBphP1* gene was cloned as described earlier^13^. PCM part encodings of the first 502 amino acids of *DrBphP* gene were PCR amplified as a NheI-KpnI fragment and cloned into the pAcGFP1-Hyg-N1 plasmid (Takara/Clontech). The *AcGFP1* gene was cut out using *Bam*HI and *Not*I enzymes and swapped with *IRES2-mCherry*, which was a fragment of the pIRES2-mCherry plasmid (Takara/Clontech). The final pIRES2-mCherry-DrBphP-PCM plasmid bears the Hygromycin resistance. The resulting plasmid allows co-expression of DrBphP-PCM and mCherry proteins from the same bicistronic mRNA. For equimolar mammalian expression from individual plasmids, the *RpBphP1* gene or the PCM part of the *DrBphP* gene were PCR amplified as BglII-AscI fragments and cloned into multi-cloning sites of the pMCS-T2A-EGFP vector developed in our laboratory. The resulting plasmids allowed equimolar co-expression of RpBphP1 or DrBphP-PCM and EGFP proteins. To obtain plasmid for equimolar expression of both BphPs in one mammalian cell, the PCM part of the *DrBphP* gene was amplified via PCR as SpeI-NotI fragment and cloned into the RpBphP1-T2A-EGFP plasmid instead of *EGFP* gene.

For mammalian expression of DrSplit, the *DrPAS* encoding DNA fragment was PCR amplified as an XbaI-XbaI fragment and cloned into the iSPLIT plasmid^28^ to generate the pC4-RHE-DrPAS. Then, the *DrPAS-FRB* encoding fragment was cut out with *Eco*RI and *Bam*HI and inserted into the multiple cloning site of the pIRES2-EGFP plasmid (Takara/Clontech) to generate the final pIRES2-EGFP-DrPAS-FRB, which bears the neomycin resistance. The resulting plasmid allows co-expression of DrPAS-FRB and EGFP proteins from the same bicistronic mRNA. *DrGAF-PHY* encoding DNA fragment was PCR amplified as a SpeI-SpeI fragment and cloned into the iSPLIT plasmid to generate pC4EN-F-DrGAF-PHY. Then *FKBP-DrGAF-PHY* encoding fragment was amplified without an NLS-signal and inserted as a NheI-KpnI fragment into the pAcGFP1-Hyg-N1 plasmid (Takara/Clontech). *AcGFP1* gene was cut out using *Bam*HI and *Not*I enzymes and swapped with IRES2-mCherry, which was a fragment of the pIRES2-mCherry plasmid (Takara/Clontech). The final pIRES2-mCherry-FKBP-DrGAF-PHY plasmid bears the hygromycin resistance. The resulting plasmid allows co-expression of FKBP-DrGAF-PHY and mCherry proteins from the same bicistronic mRNA.

For bacterial expression, the PCM encoding part of the *DrBphP* gene was cloned into a pBAD/HisB vector (Life Technologies/Invitrogen).

### Protein expression and characterization

LMG194 host cells (Life Technologies/Invitrogen) were used for protein expression. A pWA23h plasmid encoding HO from *Bradyrhizobium ORS278* (*hmuO*) under the rhamnose promoter was co-transformed with a pBAD/HisB plasmid encoding DrBphP-PCM with a polyhistidine tag. The bacterial cells were grown in RM medium supplemented with ampicillin, kanamycin, and 0.02% rhamnose at 37 °C for 6–8 h, followed by induction of protein expression by adding 0.002% arabinose and incubation for 24 h at 18 °C. The protein was purified with Ni-NTA agarose (Qiagen). The sample was desalted using PD-10 columns (GE Healthcare).

Absorption spectra of DrBphP-PCM, dissolved in phosphate-buffered saline, were measured using a standard spectrophotometer (Hitachi U-2000) with a 100 µl quartz microcuvette (Starna Cells). The spectrum of the Pr state (OFF state) DrBphP-PCM was measured without a photoswitching light source, because the OFF state was the ground state. To measure the ON state spectra, we carried out photoswitching with a 636/20 nm custom-assembled LED source placed above the microcuvette. The photoswitching beam direction was orthogonal to the optical beam path of the spectrophotometer. The ON state spectra were measured after the photoswitching was completed and the LED was turned off, so there was no interference with the measurements. Because of the extremely low-light intensity (<1 μW cm^−2^), changes in the absorption spectra of DrBphP-PCM induced by the light illumination inside the spectrophotometer were negligible. The photoswitching of DrBphP-PCM shown in Fig. 1d was performed with 780/20-nm and 636/20-nm LEDs, which were mounted orthogonally to the optical beam path of the spectrophotometer to avoid detection interference. DrBphP-PCM was photoswitched directly in the microcuvette of the spectrophotometer while its absorbance at 780 nm was measured.

FluoroMax-3 spectrofluorometer (Jobin Yvon) was used for recording fluorescence spectra. The photoswitching of the DrBphP-PCM and DrSplit fluorescence signals in cell lysates shown in Fig. 7d, e, respectively, was performed with 780/20-nm and 636/20-nm custom-assembled LED arrays. Cell lysates of HeLa cells transiently transfected with either DrBphP-PCM or DrSplit were obtained by disruption of cells by freezing-thawing. DrBphP-PCM or DrSplit were photoswitched directly in the 150 μL quartz microcuvette (Starna Cells) of the spectrofluorometer while its fluorescence at 720 nm was being measured.

### Mammalian cell culture

HeLa cells (ATCC, Cat#CCL-2) were grown in DMEM supplemented with 10% FBS, a penicillin-streptomycin mixture and 2 mM of glutamine (all from Invitrogen/Life Technologies) at 37 °C in 5% CO_2_ air atmosphere. To compare expression level of RpBphP1 and DrBphP-PCM HeLa cells were transiently transfected with RpBphP1-T2A-EGFP or DrBphP-PCM-T2A-EGFP using an Effectene (Qiagen) according to the manufacturer's protocol. Expression level of EGFP co-expressed downstream of T2A peptide with RpBphP1 or DrBphP-PCM cells was measured using a LSRII flow cytometer (BD Biosciences) equipped with a 488-nm laser and a 530/40-nm emission filter. To study the expression of RpBphP1 and DrBphP-PCM, HEK-293 cells (ATCC, Cat#CRL-1573) were transiently transfected with RpBphP1-T2A-DrBphP-PCM using Lipofecteamin 2000 (Invitrogen) according to the manufacturer’s protocol.

U87 cells (ATCC, Cat#HTB-14) were grown in DMEM supplemented with 10% FBS, a penicillin-streptomycin mixture and 2 mM of glutamine (all from Invitrogen/Life Technologies) at 37 °C in 5% CO_2_ air atmosphere. We obtained a U87 stable preclonal mixture by transfecting cells with pIRES2-mCherry-DrBphP-PCM plasmid. Plasmid transfection was performed using an Effectene (Qiagen). Cells were further selected with 75 µg ml^−1^ of Hygromycin B (Gold Biotechnology) and enriched using a FACSAria sorter (BD Biosciences) equipped with a 561-nm laser and a 610/20-nm emission filter. For further culturing of U87 cells stably expressing DrBphP-PCM, the medium was supplemented with 75 µg ml^−1^ of Hygromycin B.

MTLn3 cells (kindly provided by Dr. J. E. Segall, Albert Einstein College of Medicine) were grown in MEM Alpha supplemented with 5% FBS, a penicillin-streptomycin mixture, and 2 mM of glutamine (all from Life Technologies) at 37 °C in a 5% CO_2_ air atmosphere. The MTLn3 preclonal mixture was obtained by transfecting with DrSplit plasmids, pIRES2-EGFP-DrPAS-FRB, and pIRES2-mCherry-FKBP-DrGAF-PHY. Plasmid transfection was performed using an Effectene (Qiagen). Cells were further selected with 700 µg ml^−1^ of G418 (Corning) and 300 µg ml^−1^ of Hygromycin B (Gold Biotechnology) and enriched using a FACSAria sorter (BD Biosciences) equipped with a 488-nm laser with a 530/30-nm emission filter and a 561-nm laser with a 610/20-nm emission filter. After a week of growth, MTLn3 cells expressing DrSplit were then enriched again using a FACSAria sorter (BD Biosciences) equipped with a 635-nm laser with a 720/40-nm emission filter. Before the second enrichment, the MTLn3 cells with DrSplit were treated with rapamycin for 24 h. For further culturing of MTLn3 cells stably expressing DrSplit, the medium was supplemented with 700 µg ml^−1^ of G418 and 300 µg ml^−1^ of Hygromycin B.

For induction of the FRB-FKBP protein–protein interaction in cultured cells, rapamycin (LC Laboratories) was dissolved in ethanol and 100 nM was added to cells 22–25 h before analysis. The cells are analyzed by fluorescence spectroscopy or flow cytometry in 48 h after transfection.

To test the cytotoxicity of DrSplit, either HeLa or U87 cells were transiently transfected with pIRES2-EGFP-DrPAS-FRB and pIRES2-mCherry-FKBP-DrGAF-PHY using Lipofecteamin 2000 (Invitrogen) according to the manufacturer’s protocol. After 72 h of expression, the fluorescence intensity of EGFP and mCherry was analyzed by flow cytometry using an LSRII analyzer (BD Biosciences) equipped with a 488-nm laser with a 530/30-nm emission filter and a 561-nm laser with a 610/20-nm emission filter.

### Preparation of animals

Adult 2- to 3-month-old female nude mice (Hsd:Athymic Nude-FoxlNU, Harlan; body weight: ~20−30 g) were used for all in vivo experiments. All experimental procedures were carried out in conformity with laboratory animal protocols approved by the Animal Studies Committee at Washington University in St. Louis and the Office of Laboratory Animal Resources at California Institute of Technology. Throughout the experiment, the mouse was maintained under anesthesia with 1.5% vaporized isoflurane. The anesthetized mouse was taped to a lab-made motorized animal holder, which held the animal upright during imaging. The top of the holder was a small aluminum tube, providing anesthetic gas to the mouse, affixed to the animal’s nose and mouth, and the bottom was an aluminum cylinder attached to a permanent magnet. The magnet securely held the animal holder to the scanning stage for elevational scanning. The animal’s fore and hind legs were taped to the top and bottom parts of the holder, respectively. The two parts were connected by four lengths of 4-lb test braided fishing line (0.13 mm in diameter). The animal’s trunk was immersed in water, and its body temperature was maintained at 34 °C by circulating water through a heated bath outside the tank.

To implant xenograph tumors into the mice liver, ~10^6^ U87 cells, stably expressing DrSplit, DrBphP-PCM, RpBphP1 or un-modified, in 0.05 ml PBS were injected into mice with the guidance of a Vevo LAZR ultrasound system (Visualsonics) with a MS550D linear transducer array (Visualsonics; 40 MHz central frequency, 55% two-way bandwidth).

For induction of FKBP-FRB protein–protein interaction in vivo rapamycin was used. Rapamycin (LC Laboratories) was dissolved in ethanol to the concentration of 9 mg ml^−1^. Before its intraperitoneal injection, the concentrated stock of rapamycin was diluted in an aqueous solution of 5.2% Tween 80 and 5.2% PEG400. The injected volume was 150 µl and resulted in 4.5 mg kg^−1^ concentration.

For hydrodynamic transfection, 25 µg of each DrPAS-FRB and FKBP-DrGAF-PHY, or 25 µg DrBphP-PCM plasmids were diluted in PBS in a volume of 1 ml 10 g^–1^ body weight and injected rapidly (5–7 s) into the mouse tail vein using a 3  ml syringe fitted with a 27 gauge needle. Twenty-four hours later, mice were injected with rapamycin through the tail vein. At 40 h after the rapamycin injection, mice were anesthetized (isoflurane) and imaged using the RS-SIP-PACT system.

### Whole-body PACT using DrBphP-PCM and DrSplit

The whole-body PACT system was upgraded from our previous work^2^. In order to image DrBphP-PCM and DrSplit proteins, we combined a Ti:Sapphire laser (LS-2145-LT-150, Symphotic Tii, 20 Hz pulse repetition rate, 12 ns pulse width) and a Spitlight EVO β optical parametric oscillator (OPO) laser (Innolas Laser GMBH; 100 Hz pulse repetition rate, 4 ns pulse width). The 780 nm light from the Ti:Sapphire laser was used for both whole-body PA imaging and switching off DrBphP-PCM or DrSplit at the same time, while the 630 nm light from the Spitlight OPO laser was used for switching on the proteins. The flash lamps of the two pump lasers were synchronized, and the two lasers were individually triggered by an FPGA-based controller (National Instruments; sbRIO9626). The two laser beams were combined by a beam combiner, and their incident fluences in mJ cm^−2^ were measured by an optical power meter. The laser beam was first homogenized by an EDC-5optical diffuser (RPC Photonics), and then passed through a 130 ° conical lens (Delmar Photonics) to form a ring-shaped light pattern. The light was then passed through a home-made optical condenser to form a ring-shaped light band around the animal’s trunk. The light incident area was aligned slightly above the acoustic focal plane to ensure sufficient light diffusion. The width of the light band is ~5 mm, and its diameter was similar to the cross-sectional diameter (~2–3 cm) of a mouse. The maximum light fluence on the skin of the animal was ~2 mJ cm^−2^ at 630 nm and ~2 mJ cm^−2^ at 780 nm, respectively, which were within the American National Standards Institute safety limit exposures (20 mJ cm^−2^ at 630 nm at a 10-Hz pulse repetition rate, or 200 mW cm^−2^; 30 mJ cm^−2^ at 780 nm at a 10 Hz pulse repetition rate, or 300 mW cm^−2^)^35^.

The PA signals were detected by a 512-element full-ring ultrasonic transducer array (Imasonic; 50 mm ring radius, 5 MHz central frequency, more than 90% one-way bandwidth). Each element (0.2 acoustic numerical aperture, 20 mm element height, 0.61 mm pitch, 0.1 mm inter-element spacing) was cylindrically focused. The combined foci of all 512 elements form an approximately uniform imaging region with a ~20 mm diameter and 1 mm thickness. Within this region, the in-plane resolution was ~125 µm and the elevational resolution was ~1 mm (Supplementary Figure 2). A lab-made 512-channel 26 dB gain pre-amplifier was directly connected to the ultrasonic transducer array housing, with minimized connection cable length to reduce cable noise. The pre-amplified PA signals were digitized in parallel by a 512-channel data acquisition (DAQ) system (four SonixDAQs, Ultrasonix Medical ULC; 128 channels each, 40 MHz sampling rate, 12-bit dynamic range) with programmable amplification up to 51 dB. For image reconstruction, the raw data from each element were first deconvolved using the Wiener filter to account for the ultrasonic transducer’s impulse response, and then reconstructed within each imaging plane. To mitigate the artifacts induced by acoustic heterogeneities in the animal body, a half-time, dual-speed-of-sound variant of the universal back-projection algorithm^2,43^ was applied for reconstruction.

The CNR of the reconstructed image was calculated as the peak-to-peak PA amplitude in the region of interest (ROI), divided by two times the standard deviation of the background amplitude.

### Temporal frequency lock-in reconstruction

Thanks to the high-imaging speed of the current system, we were able to capture the entire switching process of the BphPs, which enabled temporal frequency analysis of each pixel. We extracted the amplitudes of the harmonics of the preset switching frequency (illumination modulation frequency). The highest-order harmonic was determined empirically by maximizing the CNR on the reconstructed images. This LIR method demonstrated a superior CNR over the previous differential method (Supplementary Figures 4 and 6) with the same number of switching cycles, because it averaged over the entire decay process within a cycle as well as across different cycles. The direct differential method, moreover, completely ignores the repeatability of the decay process across cycles. Typically, a threshold level of four times the noise level, estimated as the standard deviation of the background signal outside the imaged region, is globally applied to the temporal frequency domain of reconstructed PA images. To minimize the influence of respiratory motion, a non-rigid image matching algorithm^44^ was applied to the whole-body image for registration. During registration, we first selected a series of frames where respiratory motions were not obvious, and then averaged them to a single frame as a reference image. The other frames were registered to the reference image through a non-rigid image matching method.

### Calculation of decay constant

To quantify the decay constant of the switching process, raw data from multiple trials were acquired and averaged, and a sequence of PA images representing one complete decay cycle was reconstructed from the averaged data. Each PA image was smoothed by a 5-pixel-by-5-pixel square kernel to further increase the signal-to-noise ratio. The time sequence at each pixel was then fitted to an exponential decay function of the form $f\left( t \right) = a + b \cdot e^{\left( {\frac{{ - t}}{T}} \right)}$, where *t* is the time, *f*(*t*) is the measured pixel value at time *t*, *a* is the fitted signal baseline, *b* is the fitted peak pixel value, and *T* is the fitted, non-negative time constant.

When two BphPs are fully mixed in a fixed ratio inside cells, the decay function can be expressed in the form $g\left( t \right) = a + b \cdot e^{\left( {\frac{{ - t}}{{T_1}}} \right)} + c \cdot e^{\left( {\frac{{ - t}}{{T_2}}} \right)}$, where *T*_1_ > *T*_2_. Generally speaking, the absolute values of all parameters are related to the optical fluence, especially *T*_1_ and *T*_2_. Because the 1/*e* optical penetration depth for NIR light is far greater than the voxel length, we can assume that the local fluence is uniform within that voxel. Assuming that the decay rates of both phytochromes are influenced by the local fluence in the same way, the ratio *R* = *T*_1_/*T*_2_ should be relatively stable. This hypothesis was supported by our experimental results, where *R* was measured to be 2.2–3.0. The ratio $k = \frac{{{\rm{max}}\{ b,c\} }}{{{\rm{min}}\{ b,c\} }}$ should also be stable, because two phytochromes were mixed in a fixed ratio. Inside HEK-293 cells, the two phytochromes were expressed in equimolar concentrations. Indeed, the experimental results for HEK-293 cells showed that *b* ≈ *c* ≈ 0.5, and thus *k* ≈ 1.

Notice that *g*(*t*) has a more general form than *f*(*t*), thus *g*(*t*) could also be used to fit the decay curve of a single phytochrome, such as DrBphP-PCM only. Experimental results showed that *k* was much greater than 1 when only DrBphP-PCM was measured. Thus, $k = \frac{{{\rm{max}}\{ b,c\} }}{{{\rm{min}}\{ b,c\} }}$ was used as a criterion to distinguish the two types of cells—HEK-293 cells expressing both DrBphP-PCM and RpBphP1, and U87 cells expressing only DrBphP-PCM. Empirically, we determined that when 1 < *k* < 1.2, the signals were from HEK-293 cells, and when *k* > 10, the signals were from U87 cells. This criterion is independent of local optical fluence. To avoid infinity in computation, the upper limit of *k* was set to 50. During computation, a pixel-wise curve fitting operation was performed first. Then, we spatially averaged pixel values of *k* across the regions of interest, which were defined using the LIR.

If the two types of cells—HEK-293 cells and U87 cells—are mixed together and cannot be spatially separated, we can use $s = \frac{{k - 1}}{{k + 1}}$ to quantify the concentration of U87 cells, and thus $s{\prime} = 1 - s$ denotes the concentration of HEK-293 cells.

### Hematoxylin and eosin histology and fluorescence imaging

The tumor-bearing livers and kidneys and hydrodynamic-transfected livers were harvested and fixed in 4% paraformaldehyde for 24 h. After paraffin embedding, coronal sections (5 µm thick) of the livers were cut. Standard hematoxylin and eosin (H&E) staining was performed on the sections, which were examined using bright-field microscopy (NanoZoomer, Hamamatsu) with a 20 × 0.67 NA objective lens.

A lab-made fluorescence imager, including a CCD camera (DV412-BV, Andor) and camera lens (SP 272E, Tamron, 90 mm, F/2.8), was used for fluorescence imaging. A laser diode (HL6738MG, Thorlabs Inc., 690 nm, 30 mW) was used for excitation, and a bandpass filter (FB750-40, Thorlabs Inc. 750 nm, FWHM = 40 ± 8 nm) was used as an emission filter. A near-infrared LED (M780LP1, Thorlabs Inc., 780 nm, 800 mW) was used to switch the DrBphP-PCM/DrSplit proteins to Pr state, and a red LED (M625L3, Thorlabs Inc., 625 nm, 700 mW) was used to switch the proteins to Pfr state.

### Reproducibility

The experiments were not randomized. The investigators were not blinded to allocation during the experiments and outcome assessment. No sample-size estimation was performed to ensure adequate power to detect a pre-specified effect size.

### Data availability

The data that support the findings of this study are available from the corresponding authors on reasonable request. The reconstruction algorithm and data processing methods are described in detail in the Methods. We have opted not to make the data acquisition, image reconstruction, and processing code available because the code is proprietary and used for other projects.

## Electronic supplementary material

Supplementary Information

Description of Additional Supplementary Files

Supplementary Movie 1

Supplementary Movie 2

Supplementary Movie 3

Supplementary Movie 4
