# Supplementary material for: Small near-infrared photochromic protein for photoacoustic multi-contrast imaging and detection of protein interactions in vivo
Source: Nat Commun. 2018 Jul 16;9:2734. doi: 10.1038/s41467-018-05231-3 (PMC6048155; doi:10.1038/s41467-018-05231-3)
Supplement: Supplementary file 1 — Supplementary Information [file 41467_2018_5231_MOESM1_ESM.pdf]

Small near-infrared photochromic protein for photoacoustic multi-contrast imaging and detection of protein interactions *in vivo*

Lei Li, Anton A. Shemetov, Mikhail Baloban, Peng Hu, Liren Zhu, Daria M. Shcherbakova, Ruiying Zhang, Junhui Shi, Junjie Yao, Lihong V. Wang and Vladislav V. Verkhusha

Supplementary Materials

Supplementary Tables

Supplementary Table 1. Spectral and photoacoustic properties of RpBphP1 and DrBphP-PCM *in vitro*.

**Protein**	**Photo switching state**	**Maximum absorption wavelength (nm)**	**Maximum emission wavelength (nm)**	**Molar extinction coefficient (M^{-1} cm^{-1})**	**Fluorescence quantum yield (%)**	**PA excitation wavelength[a] (nm)**	**ON-to-OFF photoswitching rate[b] (s^{-1})**	**PA signal-to-noise ratio[c]**		**PA switching ratio[d]**		**Temporal frequency PA image CNR[e]**	
								0 mm depth	12 mm depth	0 mm depth	12 mm depth	0 mm depth	12 mm depth
RpBphP1	Pfr (ON)	756	none	78,300	none	780	1.56	501.4 ±15.3	15.2 ±0.3	4.3 ±0.2	2.3 ±0.2	380.3 ±12.0	21.3 ±0.2
	Pr (OFF)	678	n.d.	87,500	n.d.								
DrBphP-PCM	Pfr (ON)	750	none	59,200	none	780	0.54	636.2 ±17.2	25.4 ±1.3	8.7 ±1.5	4.7 ±1.2	550.4 ±16.0	38.3 ±0.3
	Pr (OFF)	700	720	98,000	2.9%								

[a]The laser fluence was 8 mJ cm^{-2}. The wavelengths were chosen on the basis of the absorption of the proteins and the power spectra of the lasers. [b]The ON-to-OFF photoswitching rate is defined as the reciprocal of the time when the PA signal drops to 1/*e* of its maximum, measured at [a] a laser fluence of 4 mJ cm^{-2}. [c]The protein concentration was 30 µM, and the reduced scattering coefficient of the scattering media was ~10 cm^{-1}. Data are reported as mean ± s.d. [d]The switching ratio is the ratio of the PA signal amplitudes acquired in the ON and the OFF states. Data are reported as mean ± s.d. [e]Contrast-to-noise ratio (CNR). The hemoglobin concentration was 2.3 mM. Data are reported as mean ± s.d.

Supplementary Table 2. Quantified coefficients of the decay functions of the PA signals from cells[a].

**Coefficients**[b]	**High fluence**[c]		**Low fluence**[d]		**Scattering media**[e]		
	HEK-293	U87	HEK-293	U87	HEK-293	U87	HEK-293:U87 mixture with about 1:1 ratio
a	0.014	0	0.001	0	0.035	0	0.266
b	0.493	0.968	0.515	1.01	0.500	1.00	0.169
c	0.488	0.082	0.483	0	0.481	0.0930	0.581
T_2	1.45	1.72	2.01	2.14	0.591	0.596	0.756
k[f]	1.01	11.8	1.07	100	1.04	10.8	3.44
s[g]	0.005	0.844	0.03	0.980	0.02	0.830	0.549

[a]The signals from the cells were spatially averaged first, and the calculations were based on their spatial mean values. [b]The decay function can be expressed in the form of $g(t) = a + b \cdot e^{\left(-\frac{t}{T_1}\right)} + c \cdot e^{\left(-\frac{t}{T_2}\right)}$, where $T_1 > T_2$, and the ratio $R = T_1/T_2$ is within the range of 2.2–3.0. [c]The cells were embedded in clear media, and the illumination fluence is 5 mJ/cm^2. [d]The cells were embedded in clear media, and the illumination fluence is 0.5 mJ/cm^2. [e]The cells were embedded in scattering media, and the local fluence is unknown. [f]As defined in Online Methods, $k = \frac{max\{b,c\}}{min\{b,c\}}$, which was used as a criterion to distinguish two types of cells. It can be seen from Table 2, regardless of local fluence, the k of HEK-293 cells is approximately 1, whereas the k of U87 cells is much larger, normally greater than 10, to avoid infinity in computation, the upper limit of k was set to 50. [g]As defined in Online Methods, we define $s = \frac{k-1}{k+1}$ to quantify the concentration of U87 cells if a mixture of the two types of cells was imaged.

Supplementary Figures

Supplementary Figure 1.

Development of a 1.5-fold smaller photochromic probe for differential photoacoustic imaging.

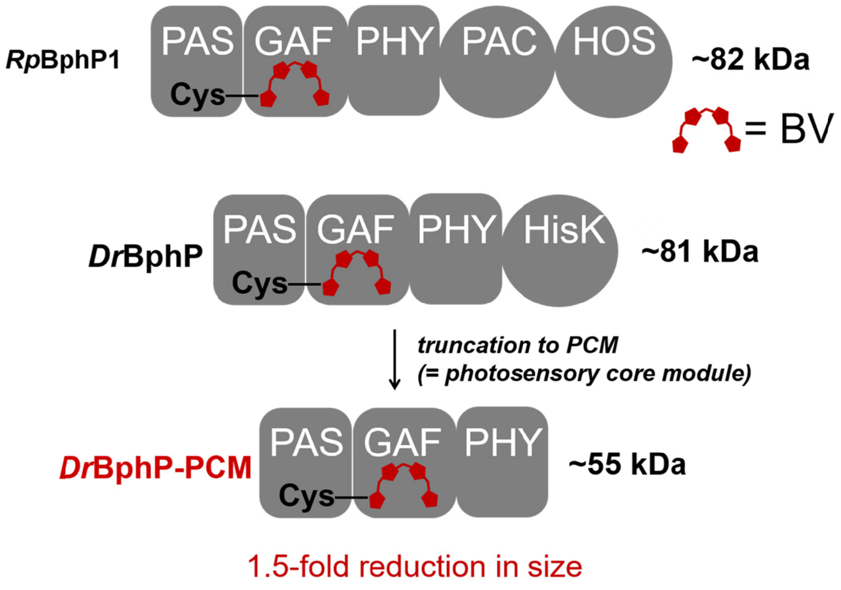

Monomers (shown) of naturally dimeric bacterial phytochromes (BphPs) share a common domain structure. It is represented here by the photosensory core module (PCM), formed by the PAS-GAF-PHY domain triad, and output domains. The biliverdin (BV) chromophore is covalently bound with conservative cysteine from the PAS domain and secured to a chromophore-binding pocket in the GAF domain. The molecular weight of the BphPs monomer is ~80 kDa. BphP1 from *Rhodopseudomonas palustris*, called RpBphP1, consists of the regular PCM and the output PAS/PAS and HOS domains. The domain organization of BphP from *Deinococcus radiodurans*, DrBphP, is comprised of the PCM and the output histidine kinase (HisK) domain. We truncated full-length DrBphP to its PCM and called the result DrBphP-PCM, achieving a 1.5-fold reduction in the molecular weight of the PA probe.

Supplementary Figure 2.

Quanfication of the spatial resolution of the RS-SIP-PACT system.

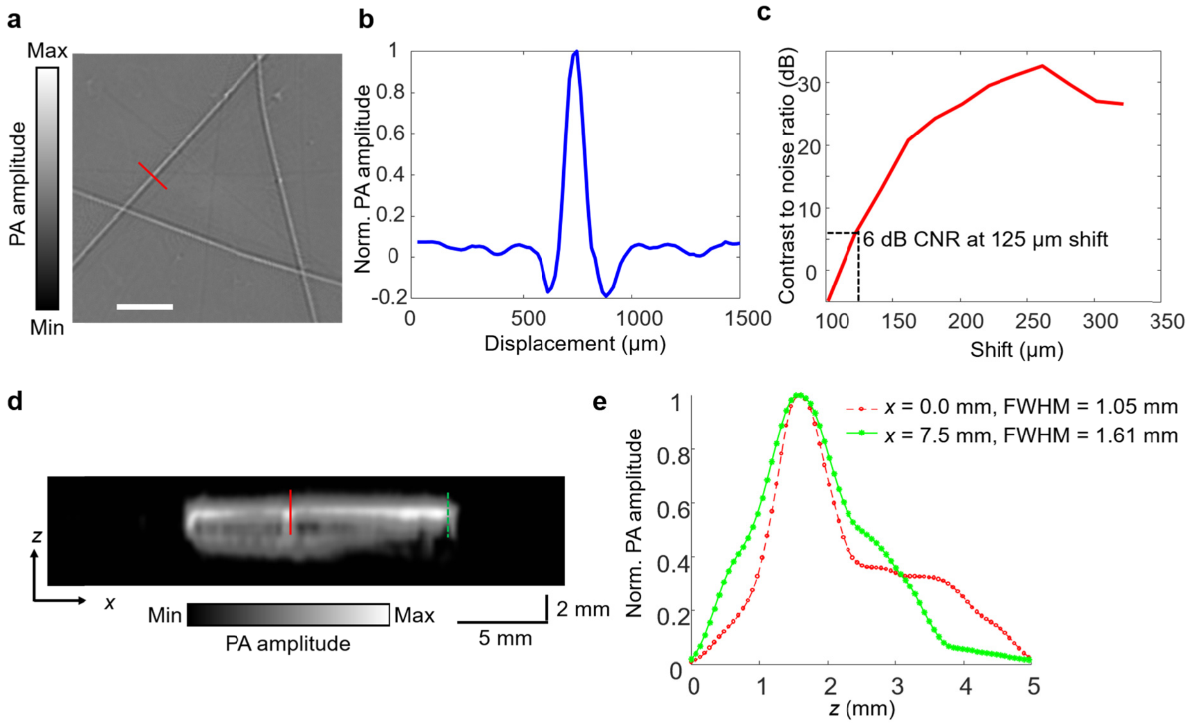

(a) An image of three tungsten wires, each with a nominal diameter of 50 μm. Scale bar, 2 mm. (b) The PA amplitude distribution along the red line in (a). (c) The contrast-to-noise ratio (CNR) versus the shift in the sum of the original line profile shown in (b) and the shifted copy. The in-plane resolution, defined as the shift corresponding to 6-dB CNR, is 125 μm. (d) The photoacoustic image of a tungsten wire with a nominal diameter of 50 μm, projected on the *x*-*z* plane. (e) The line profiles of (d) at the center of the ring (indicated in (d) by the red solid line) and at 7.5 mm off-center (indicated in (d) by the green dashed line).

Supplementary Figure 3.

Characterizations of DrBphP-PCM photochromic properties.

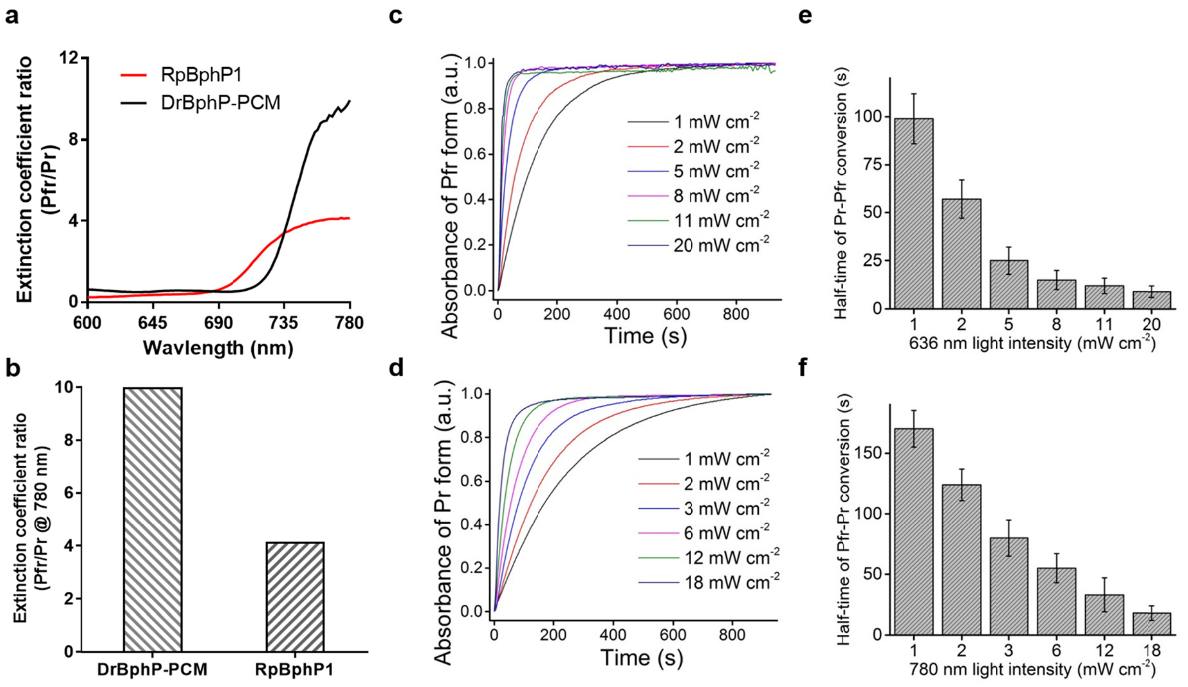

(a) Molar extinction coefficient ratios between the Pfr and Pr states of DrBphP-PCM and RpBphP1, showing the superior switching contrast of DrBphP-PCM in the NIR spectral region. (b) Molar extinction coefficient ratio between the Pfr and Pr states of DrBphP-PCM and RpBphP1 at 780 nm, showing that DrBphP-PCM has 2-times better switching contrast than that of RpBphP1 at 780 nm. (c) Dependence of the Pr→Pfr photoconversion on the 636/20 nm light intensity. The kinetic curves can be fitted with monoexponential functions ($R^2 \geqslant 0.958$ for all curves). (d) Dependence of the Pfr→Pr photoconversion on the 780/20 nm light intensity. A gradual increase in absorbance of the Pr state was observed for all tested light intensities. The kinetic curves can be fitted with monoexponential functions ($R^2 \geqslant 0.958$ for all curves). (e) Dependence of the half-time of light-induced DrBphP-PCM Pr→Pfr photoconversion on the intensity of 636/20-nm light (n = 3; error bars are s.e.m.). (f) Dependence of the half-time of light-induced Pfr→Pr photoconversion on the intensity of 780/20-nm light (n = 3; error bars are s.e.m.).

Supplementary Figure 4.

Frequency lock-in reconstruction provides better contrasts of BphPs.

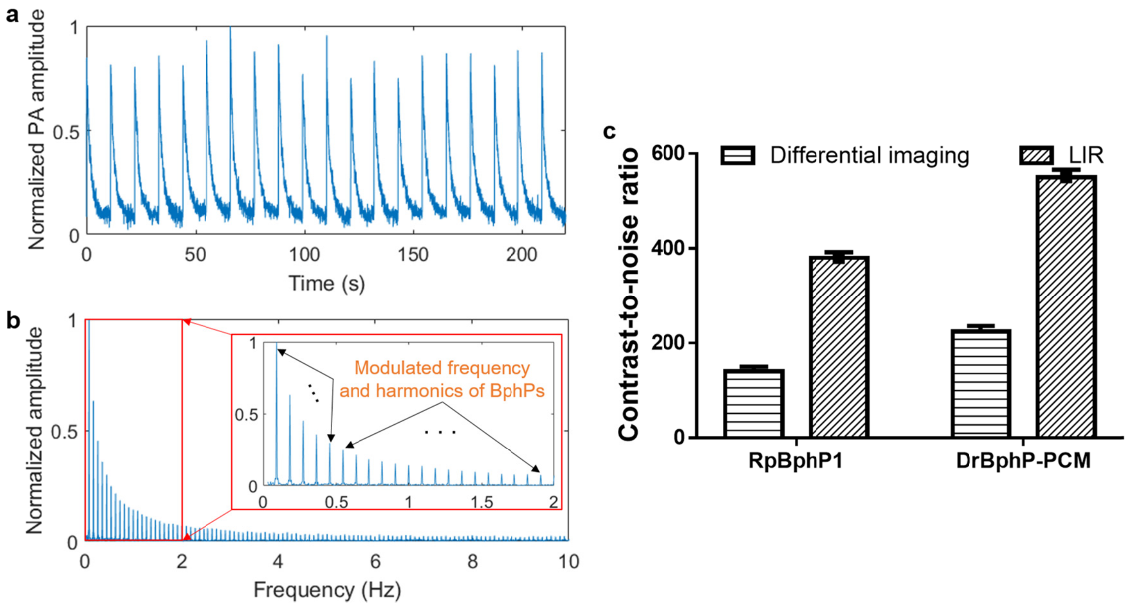

(a) Normalized photoacoustic (PA) signal of BphPs changes during the illumination modulation. (b) The temporal frequency spectrum of the signal in (a) clearly shows the modulation frequency and its harmonics. (c) Frequency lock-in reconstruction (LIR) provides ~2–3-fold better CNR of BphPs than the differential imaging method; error bars are s.e.m. (n = 40), calculated based on the pixel values from regions of interest.

Supplementary Figure. 5.

Comparison of RpBphP1 and DrBphP-PCM expression levels.

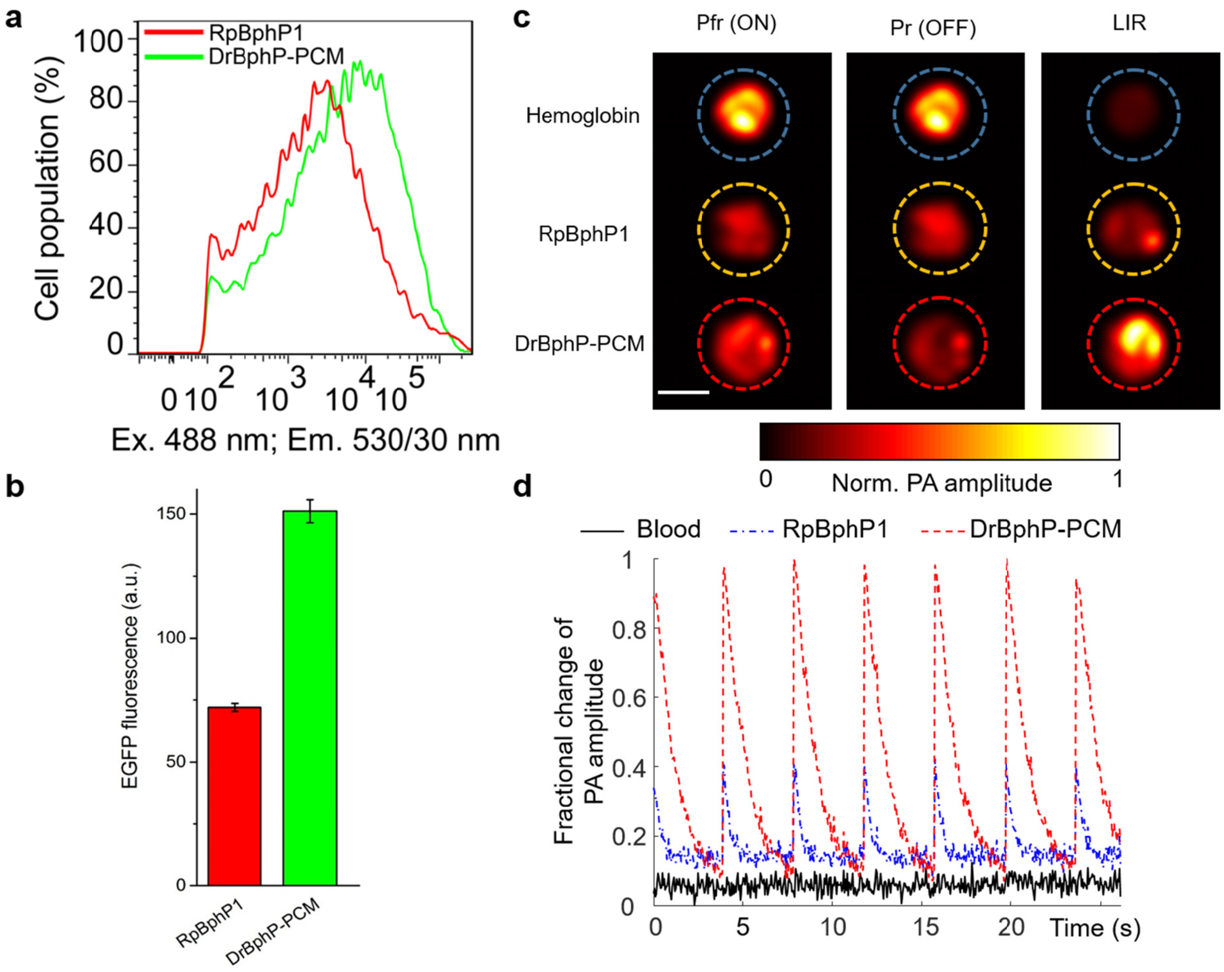

(a) Flow cytometry was used to analyze the fluorescence intensity distribution of live HeLa cells that co-expressed RpBphP1 or DrBphP-PCM with EGFP downstream of the T2A self-cleavable peptide (in BphP-T2A-EGFP constructs). The mean EGFP fluorescence intensity is presented. Error bars, s.e.m. (n = 3; transfection experiments). (b) Quantification of the data presented in (a). (c) PA images of hemoglobin, RpBphP1, and DrBphP-PCM in the Pfr (ON) state (left column) and Pr (OFF) state (middle column). LIR images of hemoglobin, RpBphP1, and DrBphP-PCM (right column). Scale bar, 2 mm. (d) Fractional change of PA signals from bovine blood, U87 cells expressing either RpBphP1 or DrBphP-PCM during 780 nm illumination.

Supplementary Figure 6.

Lock-in reconstruction of U87 cultured cells expressing either DrBphP-PCM or RpBphP1.

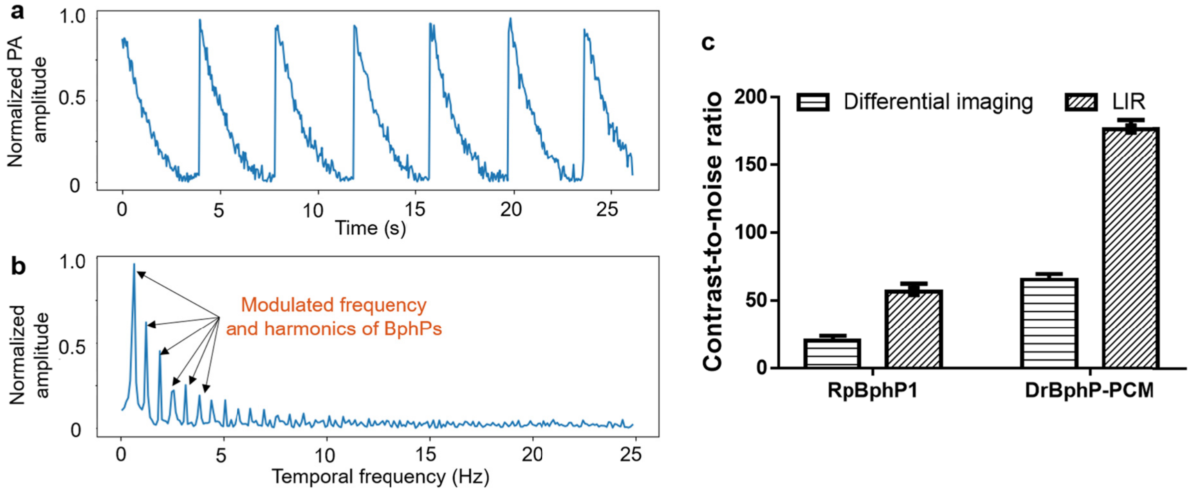

(a) Normalized PA signal of U87 cells expressing changes in either DrBphP-PCM or RpBphP1 during the illumination modulation. (b) The temporal frequency spectrum of the signal in (a) clearly shows the modulation frequency and its harmonics. (c) Frequency lock-in reconstruction (LIR) provides ~2–3-fold better CNR of U87 cells expressing either DrBphP-PCM or RpBphP1 than the differential imaging method; error bars are s.e.m. (n = 40), calculated based on the pixel values from regions of interest.

Supplementary Figure 7.

Separation of three types of cells expressing different BphPs in scattering media at different depths.

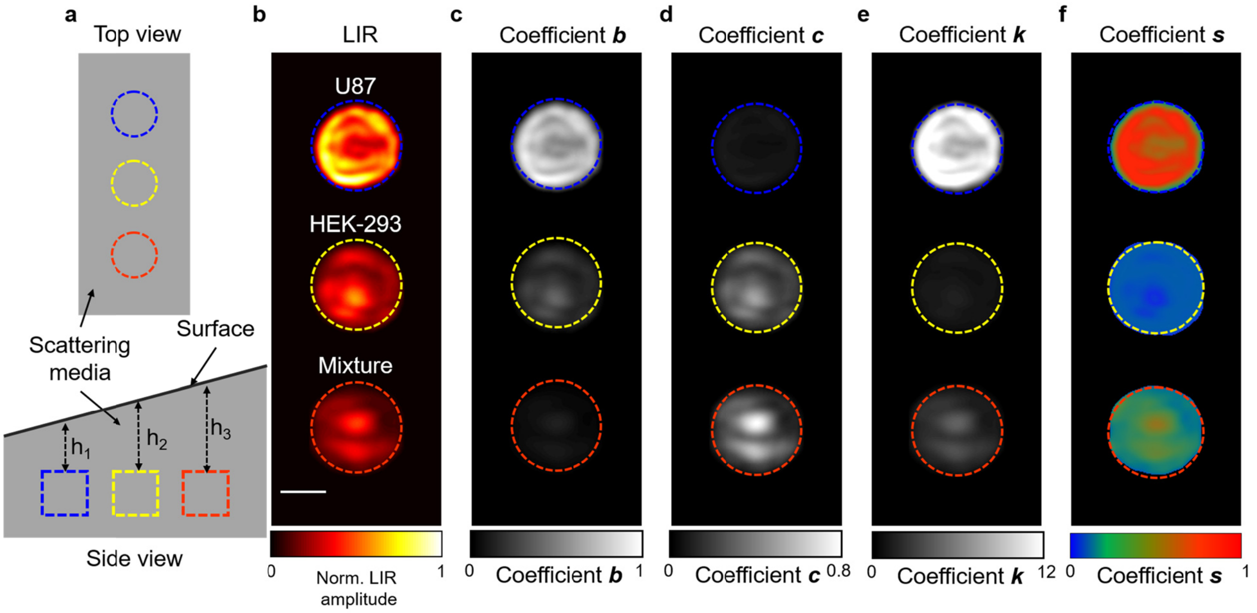

(a) Top and side views of the schematics of the phantom containing the three types of cells (top, U87 cells expressing DrBphP-PCM only, middle, HEK-293 cells expressing both DrBphP-PCM and RpBphP1, bottom, a mixture of U87 cells and HEK-293 cells), which were embedded in the scattering media with different distances to the phantom surface. Top illumination of 780 nm light was used. (b) LIR images of three types of cells. (c) Computed coefficients, *b*, of the three types of cells. (d) Computed coefficients, *c*, of the three types of cells. (e) Computed coefficients, *k*, of the three types of cells, which enables a good separation of each other. (f) Computed coefficients, *s*, of the three types of cells, which illustrates the concentrations of the U87 cells inside all cells well. Scale bar, 2 mm.

Supplementary Figure 8.

In vivo PA detection of a brain tumor expressing DrBphP-PCM.

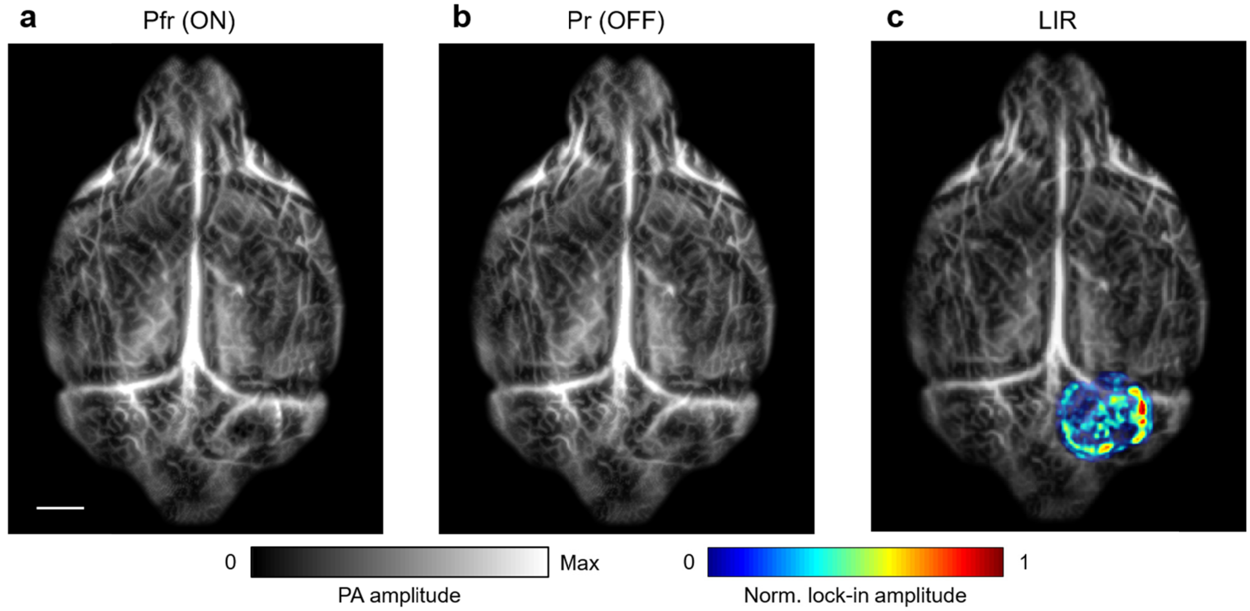

The brain of a nude mouse was imaged two weeks after the injection of U87 glioblastoma cells expressing DrBphP-PCM ~3 mm underneath the surface. PA image of the mouse brain cortex with DrBphP-PCM in its ON state (a) and OFF state (b). (c) LIR image overlaid on the brain cortex vasculature, showing the brain tumor with high contrast. Scale bar, 2 mm.

Supplementary Figure 9.

Comparison of photoswitching properties of DrSplit and DrBphP-PCM in MTLn3 cells.

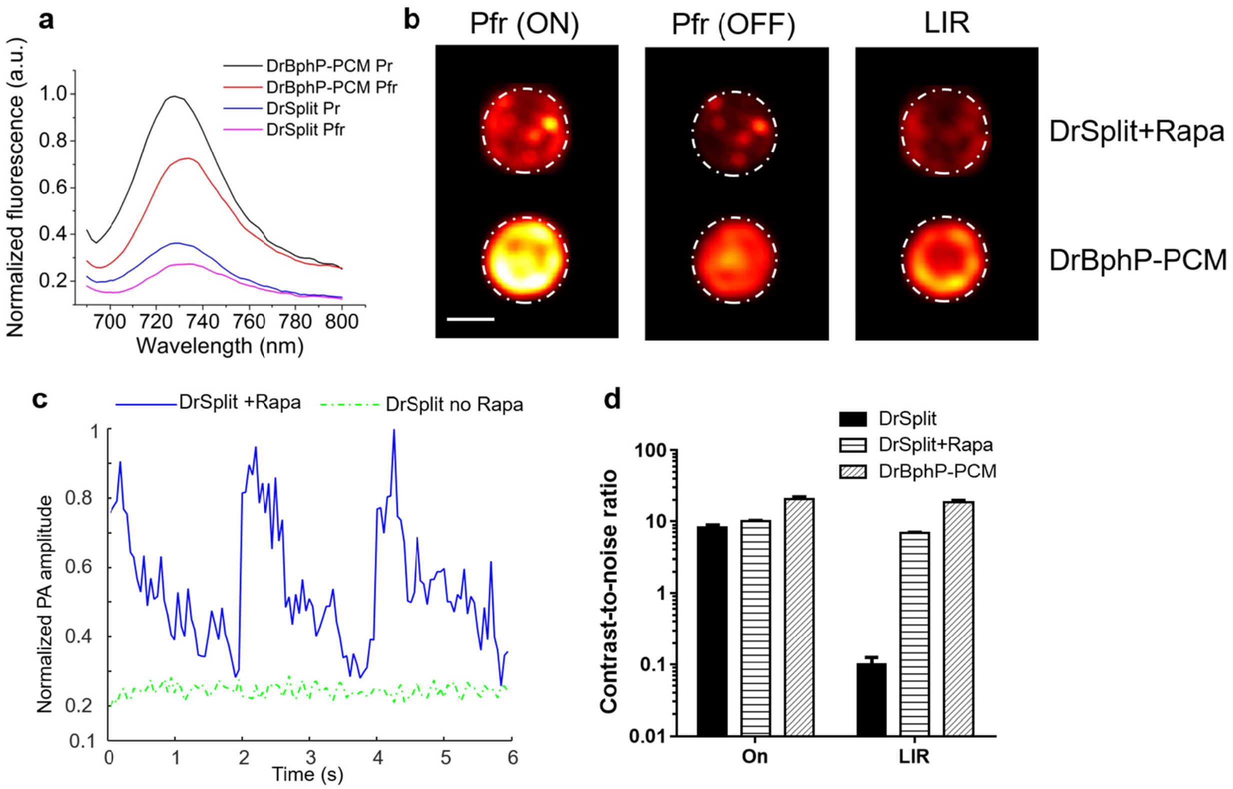

(a) Fluorescence spectra of lysates of HeLa cells expressing either DrBphP-PCM or DrSplit (treated with rapamycin). (b) ON state (left column) and OFF state (middle column) PA images of U87 cells expressing DrBphP-PCM and MTLn3 cells expressing DrSplit in the presence of rapamycin. The LIR image (right column) demonstrates that DrSplit recovered its photoswitching ability in the presence of rapamycin. In addition, DrSplit has lower switching contrast than DrBphP-PCM. Scale bar, 2 mm. (c) PA signals of MTLn3 cells expressing DrSplit in the presence of rapamycin change following the illumination modulation. In comparison, the PA signals of the controlled MTLn3 cells without rapamycin have non-detectable responses to the illumination modulation. (d) Comparison of CNRs quantified from the ON-state images and the LIR images. One sample was imaged for each type of cells expressing either DrSplit (in presence or absence of rapamycin) or DrBphP-PCM. The data were averaged over ten measurement cycles on each sample; error bars are s.e.m. (n = 40), calculated based on the pixel values from regions of interests. Rapa is short for rapamycin.

Supplementary Figure 10.

Cytotoxicity of DrSplit in mammalian cells.

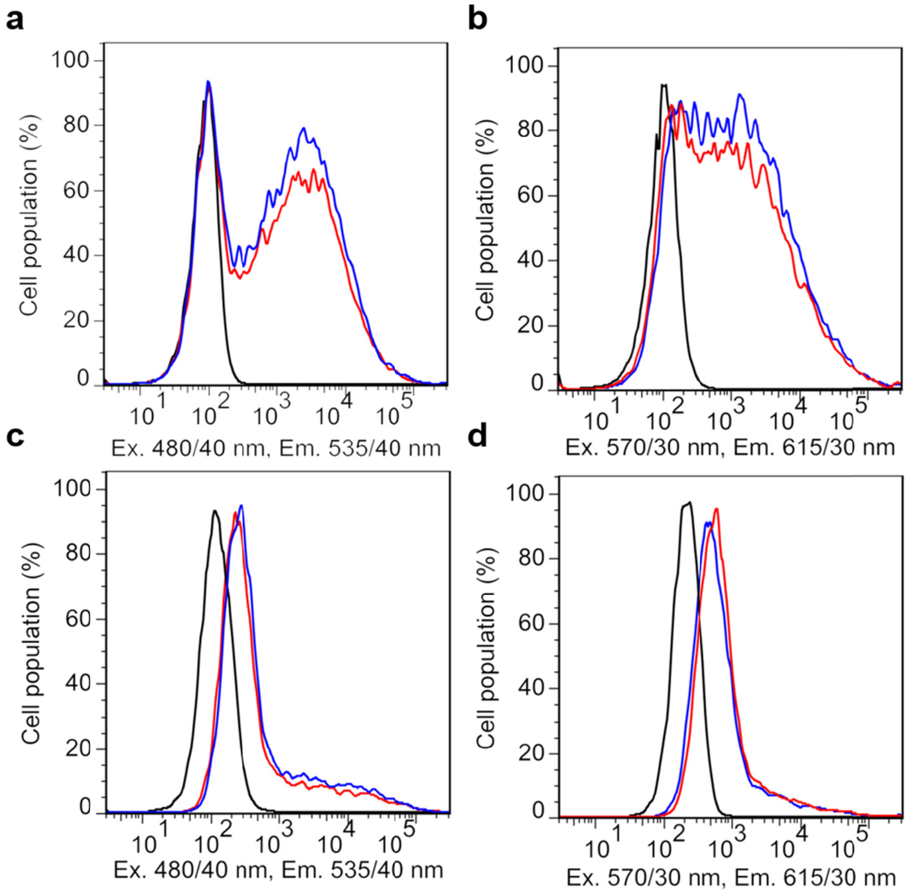

Flow cytometry was used to analyze the fluorescence intensity distribution of live HeLa (a, b) and U87 (c, d) cells expressing both EGFP and mCherry. EGFP was co-expressed upstream of IRES2 sequence from EGFP-IRES2-DrPAS-FRB. mCherry was co-expressed upstream of IRES2 sequence from mCherry-IRES2-FKBP-DrGAF-PHY. The mean values of EGFP (a, c) and mCherry (b, d) fluorescence intensity are presented. Black lines correspond to mock-transfected cells, red lines correspond to distribution of cells transfected with EGFP-IRES2-DrPAS-FRB and mCherry-IRES2-FKBP-DrGAF-PHY but non-treated with rapamycin, blue lines correspond to distribution of cells transfected with EGFP-IRES2-DrPAS-FRB and mCherry-IRES2-FKBP-DrGAF-PHY and treated with 10 nM of rapamycin.

Supplementary Figure 11.

Validation of the existence of reconstituted DrSplit via near-infrared fluorescence.

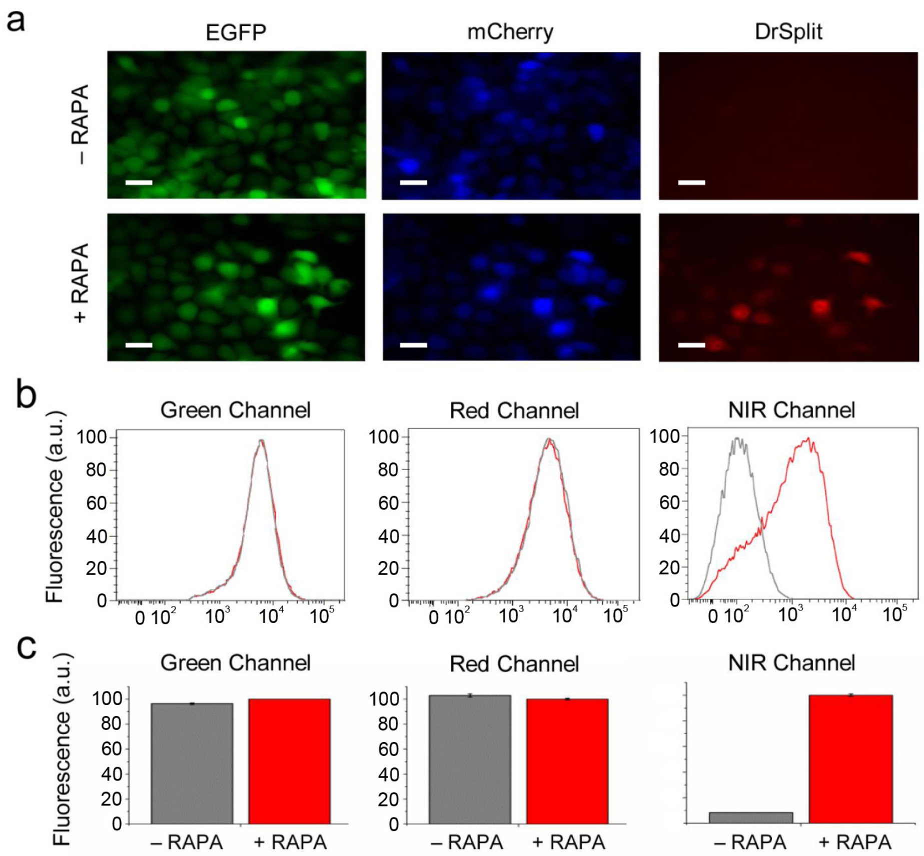

MTLn3 cells stably expressing both, EGFP-IRES2-DrPAS-FRB and mCherry-IRES2-FKBP-DrGAF-PHY. Constructs were treated with rapamycin. The fluorescence of non-treated (– RAPA) and treated (+ RAPA) cells was compared by the fluorescence of EGFP (green; ex 480/40 nm, Em 535/40 nm), mCherry (red; ex 570/30 nm, Em 615/30 nm) and DrSplit (NIR; ex 605/40 nm, Em 640/LP nm). (a) Fluorescence microscopy images of non-treated (top row) and treated (bottom row) cells. (b) Flow cytometry was used to analyze the fluorescence intensity distribution of live MTLn3 cells, that stably expressing DrBphP-PAS downstream of IRES2 sequence with EGFP and DrBphP-GAF-PHY downstream of IRES2 with mCherry. The mean values of EGFP, mCherry and DrSplit fluorescence intensity are presented. Gray curves correspond to non-treated samples, red curves correspond to samples treated with rapamycin. (c) Quantification of the data presented in (a). Error bars, s.e.m. (*n*=3; transfection experiments). Scale bars, 10 µm. RAPA is short for rapamycin.

Supplementary Figure 12.

In vivo RS-SIP-PACT of liver tumors expressing DrSplit, showing photoacoustically detected PPIs at depth.

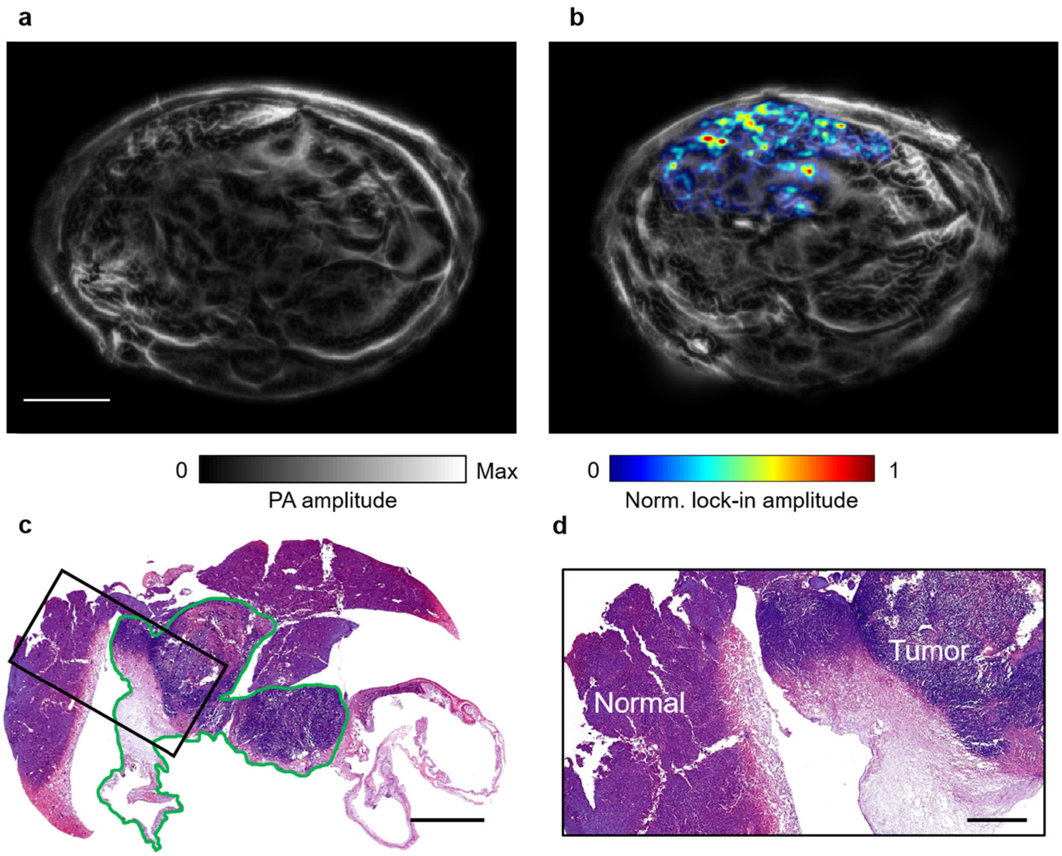

(a) PACT image of the mouse liver on Day 32 after tumor cells injection but before rapamycin injection. The LIR image was overlaid on the anatomical image, but no lock-in frequency signal was detected. Scale bar, 5 mm. (b) PACT image of the mouse on Day 34 after tumor cells injection (44 h after rapamycin injection). The LIR image was overlaid on the anatomical image, showing the PPI-induced complementation of DrBphP-PCM from DrSplit. (c) Representative H&E histological image of a harvested tumorous liver, where the tumor region is bordered by the green line. Scale bar, 2 mm. (d) Close-up of the black rectangular bordered portion of the image in (c). Scale bar, 1 mm.

Supplementary Figure 13.

In vivo fluorescence imaging of liver tumors expressing DrSplit, confirming the occurrence of PPIs.

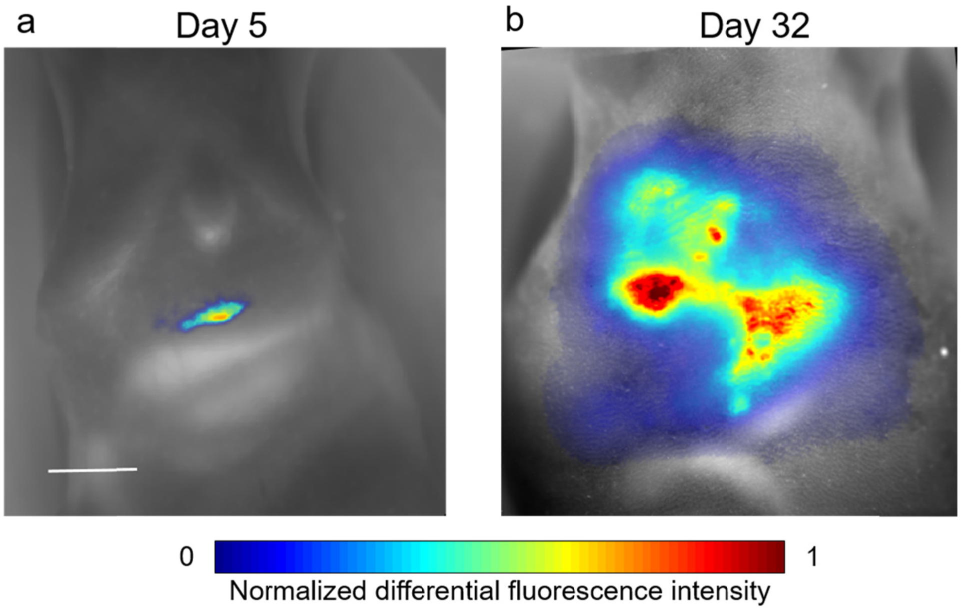

Fluorescence imaging of tumor growth and metastasis of cells expressing DrSplit. MTLn3 cells expressing DrSplit were injected in the mouse liver. Fluorescence images were acquired ~40-44 hours after injection of rapamycin through the tail vein. The differential image was computed by subtracting the Pfr state image from the Pr state image. (a) Fluorescence images of the mouse on Day 5 after the injection of tumor cells (~40 hours after rapamycin injection), the differential image (in color) was overlaid on the anatomical image (in gray). (b) Fluorescence image of the mouse on Day 32 after the injection of tumor cells (~44 hours after rapamycin injection), the differential image (in color) was overlaid on the anatomical image (in gray). Scale bar, 5 mm.

Supplementary Figure 14.

Hydrodynamic liver transfection of mice with plasmid encoding DrBphP-PCM *in vivo*.

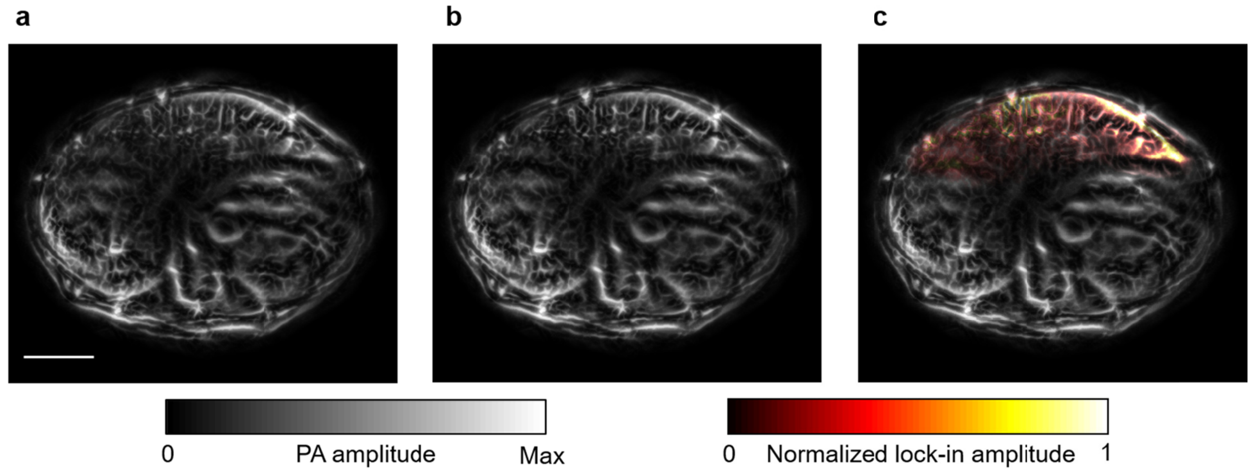

(a) ON state and (b) OFF state images of a mouse liver 24 h after injection of DrBphP-PCM plasmid. Due to the overwhelming background signal from blood, the injected plasmid-induced expression of DrBphP-PCM is not visible in either the ON state or OFF state image. (c) LIR image overlaid on the anatomical image shows the DrBphP-PCM signal in color and the background blood signal in gray. Scale bar, 5 mm.

Supplementary Figure 15.

RS-SIP-PACT of a hydrodynamically transfected mouse liver *ex vivo*.

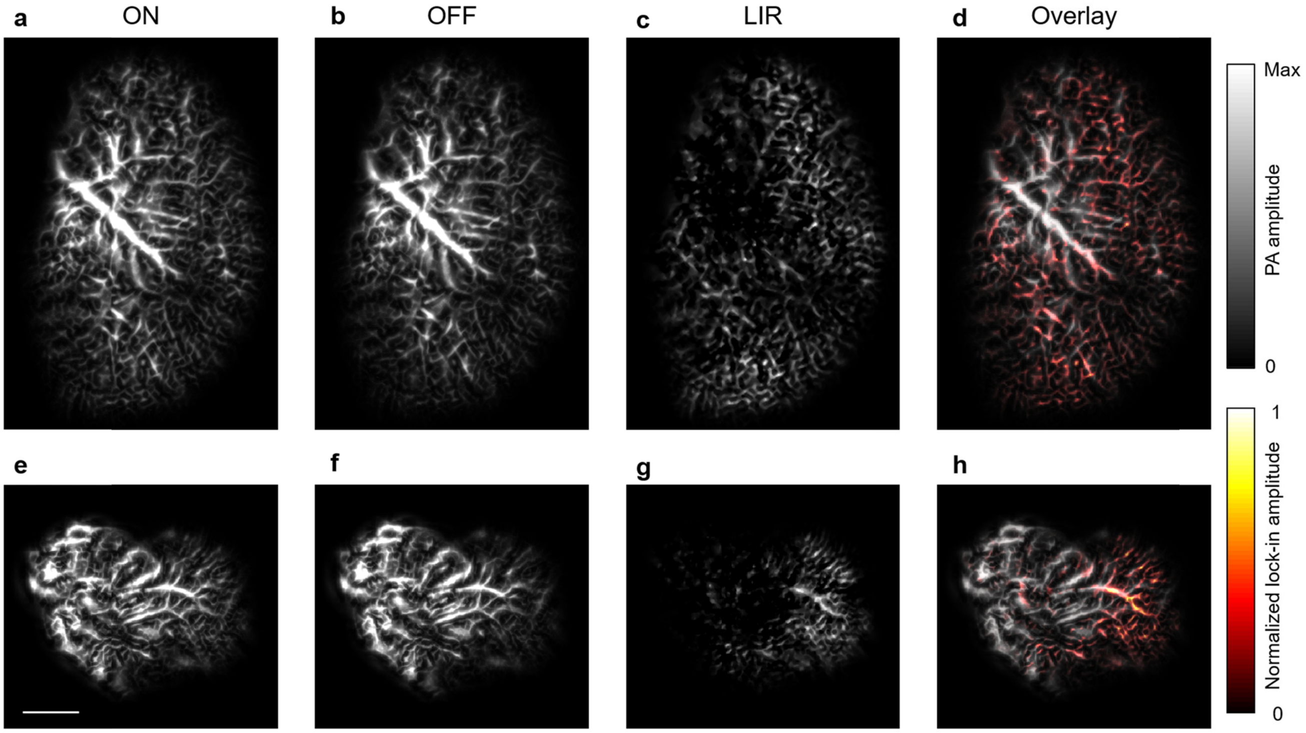

The liver was excised from a mouse after 24 hours of hydrodynamic transfection. (a) ON state and (b) OFF state PA images of the left lobe of the liver. (c) LIR image of the left lobe of the liver, selectively showing the signals from the transfection-induced expression of DrBphP-CPM. (d) Overlay image of the LIR signal with an anatomical image of the left lobe of the liver, with the DrBphP-PCM signal in color and the background blood signal in gray. (e–h) As shown in (a–e), the signals from the right lobe of the liver. Scale bar, 2 mm.

Supplementary Figure 16.

Ex vivo fluorescence imaging of the liver excised from a hydrodynamically transfected mouse expressing DrSplit.

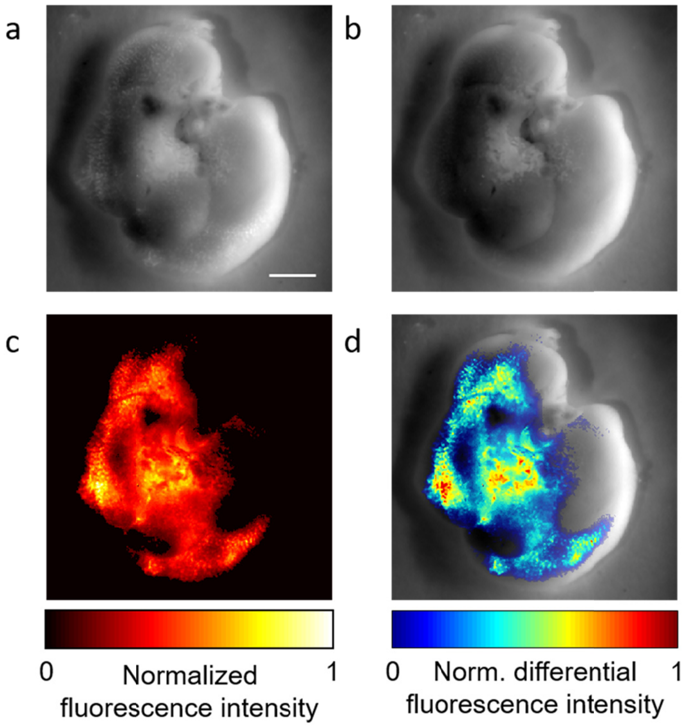

The mouse was treated with rapamycin after 24 hours of hydrodynamic transfection. The liver was excised from the mouse after 44 hours of rapamycin treatment. (a) Pr state and (b) Pfr state fluorescent images of the liver. (c) Differential fluorescent image (Pr image – Pfr image) of the liver. (d) Overlay image of differential image with the Pr state image, showing the DrBphP-PCM signal in color and the background liver signal in gray. Scale bar, 2 mm.
