# Supplementary material for: Small near-infrared photochromic protein for photoacoustic multi-contrast imaging and detection of protein interactions in vivo
Source: Nat Commun. 2018 Jul 16;9:2734. doi: 10.1038/s41467-018-05231-3 (PMC6048155; doi:10.1038/s41467-018-05231-3)
Supplement: Supplementary file 2 — Description of Additional Supplementary Files [file 41467_2018_5231_MOESM2_ESM.pdf]

Description of Additional Supplementary Files

File Name: Supplementary Movie 1
Description: RS-SIP-PACT of blood and U87 cells expressing either RpBphP1 or DrBphP-PCM clearly shows the photochromic property of the BphPs, but with different photoswitching rates.

File Name: Supplementary Movie 2
Description: RS-SIP-PACT of a tumor-bearing mouse brain highlights, through photoswitching absorption, two tumors expressing either DrBphP-PCM or RpBphP1 and reveals the different photoswitching rates of the two tumors.

File Name: Supplementary Movie 3
Description: RS-SIP-PACT of a mouse whole-body with one tumor in each kidney. The tumors are color-coded and overlaid in the 3D whole-body tomogram. Two tumors expressing either DrBphP-PCM or RpBphP1 reveal their different photoswitching rates.

File Name: Supplementary Movie 4
Description: RS-SIP-PACT of a mouse liver with DrSplit-expressing MTLn3 cells injected shows the photoswitchable signals of the tumors after the injection of rapamycin, which demonstrates the bimolecular complementation of full-length DrBphP-PCM due to the proteinprotein interaction.
